# Understanding the broader impacts of non-fatal firearm violence trauma in the United States: a scoping review

**DOI:** 10.1016/j.lana.2025.101091

**Published:** 2025-04-19

**Authors:** Deanna M. Giraldi, Susan Swingler, David S. Kirk, Sara F. Jacoby, G.J. Melendez-Torres, Elinore J. Kaufman, David K. Humphreys

**Affiliations:** a Department of Social Policy and Intervention, University of Oxford, Barnett House, 32 -37 Wellington Square, Oxford OX1 2ER, UK; b Department of Criminology, University of Pennsylvania, McNeil Building, 558, 3718 Locust Walk, Philadelphia, PA 19104, USA; c Department of Family and Community Health, University of Pennsylvania School of Nursing, Claire M. Fagin Hall, 418 Curie Boulevard, Philadelphia, PA 19104-4217, USA; d Faculty of Health and Life Sciences, University of Exeter, St Luke’s Campus Heavitree Road, Exeter EX1 2LU, UK; e Division of Traumatology, Surgical Critical Care, and Emergency Surgery, University of Pennsylvania Perelman School of Medicine, 3737 Market St 4th Floor, Philadelphia, PA 19104, USA

**Keywords:** Firearm violence, Trauma, Exposure, Survivors, Burden, Guns, Non-fatal, United States

## Abstract

Exposure to firearm violence produces ripples of impact that extend far beyond the physical wounds of direct survivors. This scoping review aimed to describe the breadth of the last 25 years of literature on short-term, long-term, and cumulative impacts of firearm violence in the United States across physical, psychological, social, and economic domains. We searched PubMed, Embase, Scopus, PsycINFO, CINAHL, ProQuest Social Science Premium (ASSIA, NCJRS, and ERIC) and Web of Science until March 2024. Among 3172 articles, 87 met inclusion criteria. Our findings suggest that research most often explores short-term and psychological impacts on direct survivor-witnesses. The review highlights notable gaps, particularly regarding long-term and cumulative impacts among both the immediate social networks of survivor-witnesses and their wider communities. Further research is necessary for the effective development of trauma-informed interventions and the provision of economic resources aimed at supporting a growing population of survivors and communities affected by violence.

## Introduction

Every year, more than 40,000 Americans are killed in acts of firearm violence, and approximately 85,000 more are shot and wounded.^1^ In addition to survivors with physical injuries, there is a multitude of individuals affected by firearm violence, either as witnesses or through their relationships with family or other community members.^2^ These individuals and communities often live with consequences of violence that span physical, psychological, social, and economic spheres, often in combination.^3^ Thus, the impacts of firearm violence extend far beyond the statistics that dominate national debates and daily media coverage.

Non-fatal firearm injuries occur more than twice as often as deaths from firearm injuries.^4^ Survivors have reported increased chronic pain, new functional limitations, reduced physical and mental health composite scores, high rates of post-traumatic stress disorder, lower employment and return to work rates, poor social functioning, and increased alcohol and substance abuse.^5^, ^6^, ^7^, ^8^, ^9^ Despite these reports of the implications of firearm violence on lived experience, research must explore the broader, ongoing impacts of firearm violence for survivors, their immediate social networks, and wider communities with shared identities, particularly geographic proximity in neighborhoods that experience disproportionate rates of violence.

In a national poll, 59 percent of adults in the United States reported that they or someone they care for had experienced firearm violence in their lifetime.^10^ Adult homicide survivors often experience poor physical and psychological outcomes, a decrease in cognitive abilities, and damaged friendship structures with exposure to firearm violence disproportionately concentrated in poor communities of color.^11^ Likewise, youth indirectly exposed to community violence exhibit poor mental health outcomes to varying degrees depending on physical proximity to the shooting and time since the event.^12^

Firearm injuries of all types (e.g., peer and partner violence, mass shootings, suicide and self-harm, and unintentional injury) are associated with high rates of mental and physical harm for children and adolescents.^13^ Each year, these injuries lead to approximately 30,000 initial in-patient hospital stays, with each stay costing around $31,000. In other words, the annual economic cost of initial physical treatments for firearm injuries is an estimated $1 billion.^14^ Repeated, or cumulative, exposures to firearm violence have the potential to reinforce both the traumatic experiences and linked outcome burdens for individuals and communities; therefore, this cost analysis may be an underestimate, illuminating a critical theme in firearm violence—disproportionate attention to short-term outcomes.

The National Trauma Research Action Plan Scoping Review explored the long-term impacts of firearm violence on survivors across the United States.^3^ Similar reviews addressed youth exposure to firearm violence, noting a need to conceptualize the ‘multiple dimensions of firearm violence exposure’.^12^^,^^13^ Sorenson and Schut investigated non-fatal firearm use in intimate partner violence, underscoring a need to expand homicide research to include the impact non-fatal usage of firearms for control.^15^ Notably, these reviews focus on mass shootings, emphasize prevention, and highlight a lack of evidence regarding cumulative impacts of firearm violence.

This scoping review aims to assess the breadth of research that encompasses witnesses of violence, their families, and communities with shared identities in addition to wounded survivors. Unlike previous reviews, we aim to examine short-term, long-term, and cumulative impacts of exposure to a range of firearm violence (e.g., mass shootings, interpersonal violence) on individual, interpersonal, and community levels. The following research question was formulated: How have researchers conceptualized the harms of firearm violence over the last 25 years?

## Methods

We conducted our scoping review based on a prospectively registered protocol (https://doi.org/10.17605/OSF.IO/M3S79) and reported it in accordance with the PRISMA Extension for Scoping Reviews (PRISMA-ScR).^16^^,^^17^

### Search strategy and selection criteria

We searched seven databases until March, 2024—PubMed, EMBASE, Scopus, PsycINFO, CINAHL, ProQuest Social Science Premium (ASSIA, NCJRS, and ERIC) and Web of Science Core Collection—for English-language articles published after the April 1999 mass shooting at Columbine High School that describe a range of outcomes (e.g., physical, psychological, social, and economic) of firearm violence on survivors, witnesses, and wider communities. We created the original search in PubMed and subsequently adapted it to the additional databases (see Supplementary Methods for the search strategy across databases). To enhance the search scope, we also reviewed grey literature, namely published dissertations and reference lists of included articles.

We included any full-length empirical (quantitative, qualitative, or mixed-methods study) or non-empirical (scoping reviews) articles focusing on the impacts of firearm violence on exposed individuals and communities in the United States. Like Ranney et al. (2019), we defined “exposure” broadly as a personal injury or experience of injury of another person by a firearm, including witnessing the firearm tragedy directly or belonging to the same community as someone affected. Articles describing non-lethal impacts of firearm violence, including interpersonal violence, unintentional shootings, and mass shootings were included; however, we excluded articles with “violence” measurements that did not disaggregate the impacts of firearm violence. Impacts of firearm violence spanned physical injury, psychological and social changes, shifts in quality of life, and economic burdens.

We included only articles published after the 1999 mass shooting at Columbine High School. As a watershed event, Columbine catalyzed a national reckoning with the prevalence of shootings, resulting in increased scholarly focus on the underlying causes, societal impacts, and policy implications of gun violence.^18^ This time frame ensures that the findings are grounded in the modern context of firearm-related discourse and legislation, reflecting the contemporary socio-political and cultural shifts in understanding firearm violence and thereby enhancing the relevance and applicability of the insights generated.

We excluded anatomical wound evaluations and surgical interventions to ensure that our analysis remained centered on broader physical impacts on civilians post-firearm violence exposure rather than on acute clinical management. We also excluded suicide reports, shooting events outside of the United States, policy studies, non-English articles, studies focusing on war-related consequences, air-soft or BB guns, and firearm injuries beyond the civilian context (e.g., legal intervention).

### Article selection and data extraction

We initially screened the identified articles’ titles and abstracts using Covidence. Authors involved in this phase piloted the screening guidance by reviewing the same 15 titles and abstracts. Two authors (DG & SS) then screened the remaining titles and abstracts, and any uncertainties about a record's exclusion led to a discussion with another author. We adopted the same process for full text review.

**Fig. 1:**
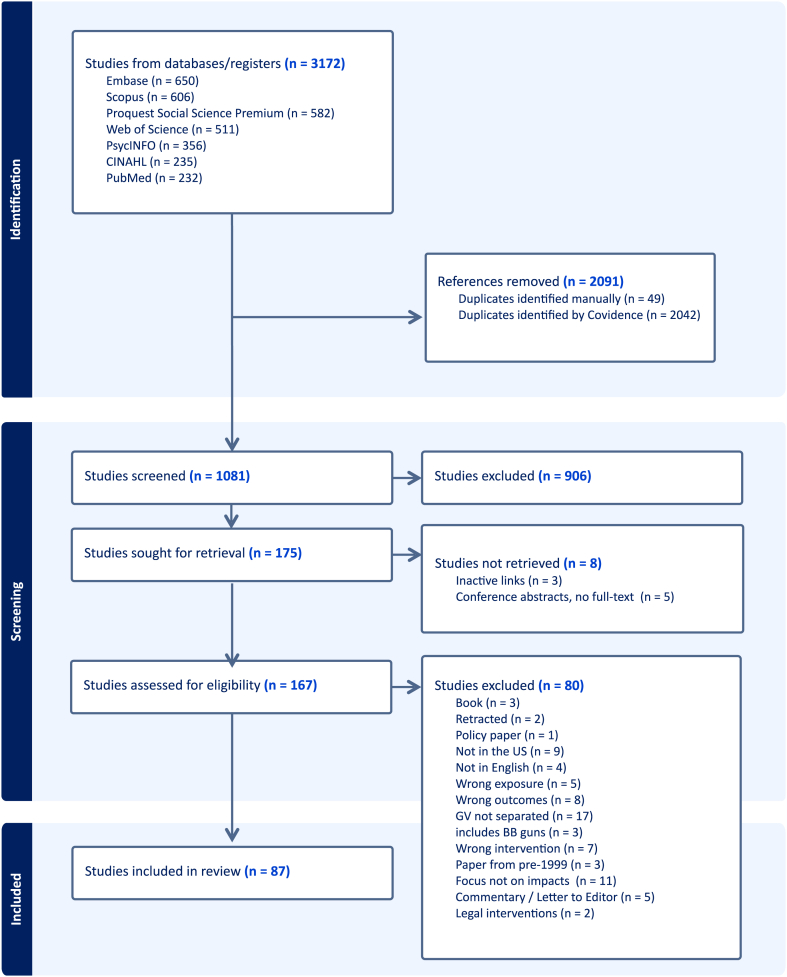
PRISMA-ScR flow chart.

Two reviewers (DG & SS) jointly developed a data extraction form in Covidence to determine which variables to extract. One author (DG) performed the data extraction, with a second author (SS) verifying all entries. The extraction form captured the following data fields: publication year, authors, aim of study, design, population description, type of firearm violence exposure, type of outcome, timeframe of outcome considered, main findings, limitations and notable strengths, funding, and conflicts of interest. We extracted this information for each article under full-text review, and mapped a flow chart of retained and excluded materials (Fig. 1) utilizing the PRISMA-ScR guideline.

### Synthesis methods

We categorized each study based on the primary domain emphasized in the study’s objectives, results, or discussion. When a study addressed multiple domains without a clear primary focus, we assigned it to the domain most prominently featured in the analysis. To avoid duplication, each study was counted only once within the results; however, in cases where a study contributed meaningfully to more than one domain, we noted these cross-domain findings in the narrative synthesis. Studies categorized within the “Quality of Life” (QoL) domain specifically utilized QoL measurements.

**Fig. 2:**
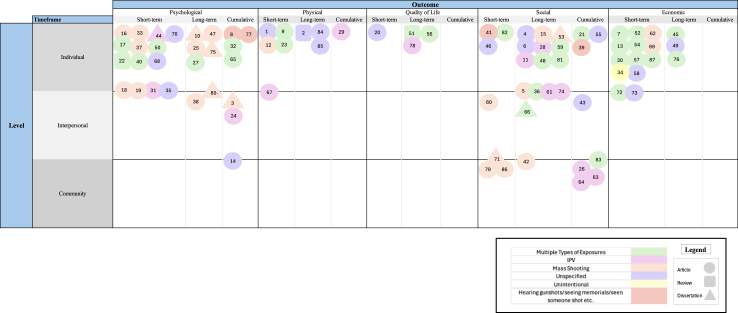
Visual summary of evidence and gaps in the literature. A list of studies corresponding to each number can be found in Supplementary Fig. S1. Note, Unspecified = studies that did not desegregate types of firearm violence (e.g., interpersonal violence, OIS, mass shooting, unintentional) or multiple types of exposure = did not define what type of firearm violence was explored. Each shape (squares refer to reviews and triangles designate dissertations) represents an individual study; the color of each shape indicates the type of firearm violence explored. The placement underscores the domain of the research (psychological, physical, quality, of life, social, or economic) as well as the temporality (short-term, long-term, or cumulative) and people impacted.

We created a visual summary of evidence (Fig. 2) to synthesize the data, showing each included article within a three-dimensional matrix of outcome (psychological, physical, quality of life, social, and economic), temporality as ‘short-term’ (i.e., up to one year from the point of firearm violence exposure), ‘long-term’ (i.e., one year or more since the exposure), and ‘cumulative’ (i.e., repeated, compounded exposures); and socioecological level as ‘individual’ (i.e., any personal impact of firearm violence, whether direct (e.g., being shot) or indirect (e.g., witnessing a shooting)), ‘interpersonal’ (e.g., ripple effects on their families, caretakers, or immediate networks), and ‘community’ (e.g., harms to those with geographical proximity). As the purpose of a scoping review is to map available evidence, we did not conduct any risk of bias assessment or quality appraisal of included studies. This approach aligns with the methodology outlined by Munn and colleagues.^16^

We grouped articles by outcome to ensure that mixed methods were reported comprehensively, giving attention to both qualitative insights and quantitative findings by adopting aspects of both the ENTREQ and SWiM guidelines, respectively.^19^^,^^20^ ENTREQ guided contextual understanding and thematic synthesis, while SWiM guidelines informed the incorporation of descriptive statistics and effect direction, accounting for trends where the data is too heterogeneous for a traditional meta-analysis. Consistent with scoping review methods, we used tabular and thematic strategies to summarize the extracted data.^16^^,^^17^ The effect directions of each study were categorized as positive, negative, or neutral based on the reported outcomes. We then mapped each effect direction to the relevant themes identified during the thematic synthesis, providing a nuanced understanding of how these effects varied across different outcomes.

## Results

After removing duplicates, we retained 1081 articles for review by title and abstract for potential inclusion (Fig. 1). Of these, 167 abstracts moved forward to full text review. After full text review, 87 articles met full eligibility criteria and were included in the final sample for the scoping review.

Among included articles (n = 87), 44.8% (n = 39) used cohort study designs to examine the impact of firearm violence exposure; of the remaining studies, 7% (n = 6) were case–control studies, 19.5% (n = 17) cross-sectional studies, and 28.7% (n = 25) qualitative studies. Eight of the studies (9.2%) were grey literature in the form of dissertations; the other 79 (90.8%) were peer-reviewed journal articles. We categorized all included studies based on the outcomes analyzed, the type of firearm violence exposure, the populations affected, and the timeframe over which the outcomes were measured, visually summarizing evidence and gaps in literature (Fig. 2).

Approximately half of the total studies (52.9%, n = 46) examined “multiple types of exposure” or “unspecified” categories of firearm exposure, meaning that these studies did not desegregate types of firearm violence or did not define what type or types of firearm exposure were explored, respectively. Twenty-three studies (26.4%) described exposure to mass shootings, thirteen (14.9%) discussed interpersonal violence, and one article centered on unintentional shootings (1.1%). Only four studies highlighted indirect exposure to firearm violence (e.g., hearing gunshots, seeing street memorials, or seeing a deceased person) (4.6%). Approximately one-third of studies (32.2%) centered on psychological impacts of exposure; sixteen (18.4%) investigated economic impacts; four (4.6%) presented quality-of-life measures.

Two-thirds (n = 58) of the included studies utilized quantitative methods, twenty-five studies (28.7%) employed qualitative methods, and four (4.6%) reported mixed methods. In Box 1, we present a synthesis matrix of our included studies; the qualitative insights of included studies are presented alongside quantitative findings to highlight matches and gaps. Supplementary Table S1 provides further details for each of the 87 included studies including aim of the study, study design, and main findings.

Box 1 **Synthesis matrix.**

**Table:** 

Qualitative insights	Quantitative findings
**Isolation** caused by physical and social restriction due to **fear** is particularly common in urban areas and may lead to negative coping mechanisms including gun carrying or avoidance of medical services as a result of distrust.^12^^,^^38^ • *“It made me think that anything can happen at any given time. Anything. So, I just created my own little circle and my own little zone and stayed in it. That's what I did.”* • *“I could stay in the house and never come out never again; or, I gotta protect myself … If I had my gun, I probably woulda shot him before he shot me. You know?”*	• Urban youth reported more violence exposures than their non-urban counterparts, including hearing gunshots (69% vs. 19%, respectively), witnessing a shooting (24% vs. 6%), and witnessing an arrest (58% vs. 27%).^53^ • The odds of gun carrying were increased by approximately 43% in recall periods for men with a history of criminal offending.^54^ • Witnesses of gun violence were no more or less likely to demonstrate a propensity for carrying a handgun across adolescence and into adulthood compared to those who did not witness gun violence after controlling for theoretical covariates (β = 0.015, 95% CI = 0.022, 0.052).^55^ • Among exposed youth, 50% took protective action to keep themselves safe, and 58% reported being very or extremely afraid, sad, or upset as a result of the indirect gun violence. More youth living in urban compared with non-urban areas took some protective action.^39^ • The significant decrease in the high-risk behavior of firearm weapon carriage at 3–6 months post-injury suggests that there is an important post-injury “teachable moment” that should be targeted with preventive interventions.^40^
• Survivors note the importance of their family and social networks as their main source of **emotional support**.^31^, ^32^, ^33^, ^34^, ^35^ • *“My friends have really showed up in all sorts of ways I never would have imagined. They are the reason I keep going. I could see that I’m important to them and they are important to me.I took 2 weeks off work and they paid me, and my boss visited me at the hospital … I just had so much support.”* • Being able to publicly **tell the stories** of the loss of loved ones is a critical part of the healing process when dealing with a traumatic loss.^56^, ^57^, ^58^ • *“We need to embrace their memories.”*	• Student survivors reported on participation in and helpfulness of organized events in the first weeks post-tragedy: 35.3% participated in candlelight vigils, 14% in “Not One More” rallies, 59.1% in the campus memorial service, 51.8% in class discussions about the event, and 43% in community-building activities.^51^
Perceptions of **manhood**, loss of independence and a perceived burden on others, and lessened mobility post-injury characterize the psychological and physical experience of the violent aftermath for Black men.^58^^,^^59^ • *“I’m so used to always being able to get up, go out the door and come back with whatever we need, whether it’s food, rent money, you know? It’s been taken away from me … Now … I’m dependent on everybody around me for everything. So now, I just stay at one spot, don’t move around too much this way. I don’t work up an appetite. I feel like a burden.”*	• Black race and firearm injury were associated with more than 3-fold higher likelihood of repeat injury compared to white race after adjusting for age, sex, insurance, and child opportunity index.^60^ • Males ages 18 to 64 have more than double the percentage of all disability types in high shooting rate neighborhoods compared to low rate neighborhoods; social isolation, chronic illness, unhealthy behavior, and poverty were consistently associated with higher prevalence of community disability irrespective of age.^61^
**Linguistic and cultural competence** is critical to responding to acts of firearm violence that target racially-marginalized communities.^42^^,^^57^^,^^62^ • In reference to 98% of survivors and families from Pulse receiving services in Spanish: *“That’s their major language, that is how they express their feelings.”*	
**First responders, clinicians, and staff,** particularly those who share the ethnic background of those wounded, bear the brunt of trauma, second only to the families and communities that suffer the loss of loved ones.^57^^,^^63^, ^64^, ^65^ • *“It is 5 AM. Page reads: ‘29-year-old male, GSW to the head ETA 10 min’ I walk to the trauma bay delirious, rubbing the crust between my eyes. The patient comes in. Paramedics rattle off information. I approach the patient and look down at his face. It is me.”* • *“Every time I hear about a school shooting in the news, anything like that, I just remember [my patient] in my bay.”*	• More than 50% of violence interventionists reported (at least rarely) experiencing 9 of the 17 STSS (Secondary Traumatic Stress Survey) items; further, only 6% of the interventionists reported experiencing *none* of the STSS items in the last 7 days, indicating that 94% of workers experienced at least one of the STSS items in the last 7 days.^66^
The disconnect between **law enforcement** and survivor families makes the grieving process more difficult as direct information, support, and closure are often lacking.^67^ • *“But I've never got any kind of closure, like from the police, they never came and said ‘Well Miss we got him, we caught him’ or this and that and then everything else was happening and I didn't forget … They never did that for me.”*	
Gun ownership and gun access, unsafe storage, direct and symbolic **threats**, and financial and immigration-related concerns exacerbate the harms of **domestic violence** with firearms.^68^^,^^69^ • *“He's also come to my car with a fake gun. Which I know what that meant. That's a real subtle sign of I can kill you. The judge wouldn't think anything of it. But I know what his cues are.”* • *“He could very well leave one of those weapons in my vehicle and turn things around by alleging that I purchased that weapon with the sole intent of physically harming him … and there’s no defense.”*	• Women who witnessed violence are more likely to experience physical and mental health problems with direct associations being 0.06 and 0.042, and those in poor physical and mental health in turn are at higher risk of experiencing housing instability with direct associations being 0.065 and 0.155, respectively.^70^
**Financial** struggles in the aftermath of firearm violence complicate the already difficult experience of injury.^36^ • *“Getting shot has caused all sorts of hassles. I have to do everything by myself so it’s hard to do things when I can’t move, making it hard to go to work. I have had a lot of medical bills and have no kind of insurance.”* • Survivors universally noted the importance of their family and social networks, and having financial safety-nets through health insurance, victims of crime assistance, and other financial assistance programs.^33^ • *“Just cause of my job, I had good insurance. Most people don’t have good insurance. That was one thing I didn’t have to worry about. I didn’t have to worry about paying and a lot of that stress was taken off.”*	• After nonfatal firearm injury, medical spending increased $2495 per person per month (402%) and cost sharing increased $102 per person per month (176%) among survivors relative to control participants (P < 0.001) in the first year after injury.^44^ • Children and adolescents’ health care spending increased by an average of $34,884—a 17.1-fold increase—with 95 percent paid by insurers or employers.^45^ • In the Northeast, hospital costs were $1.98 billion (13.9% of total), of which 56.0% was covered by government payers; for the Midwest, costs were $1.53 billion (19.7% of total), 40.4% of which was covered by government payers; in the South costs were highest at $3.2 billion (41.4% of total), but government payers only covered 34.3%; and costs for the West were $1.94 billion (25.0% of total), with government programs covering 41.6% of the cost burden.^50^

**Fig. 3:**
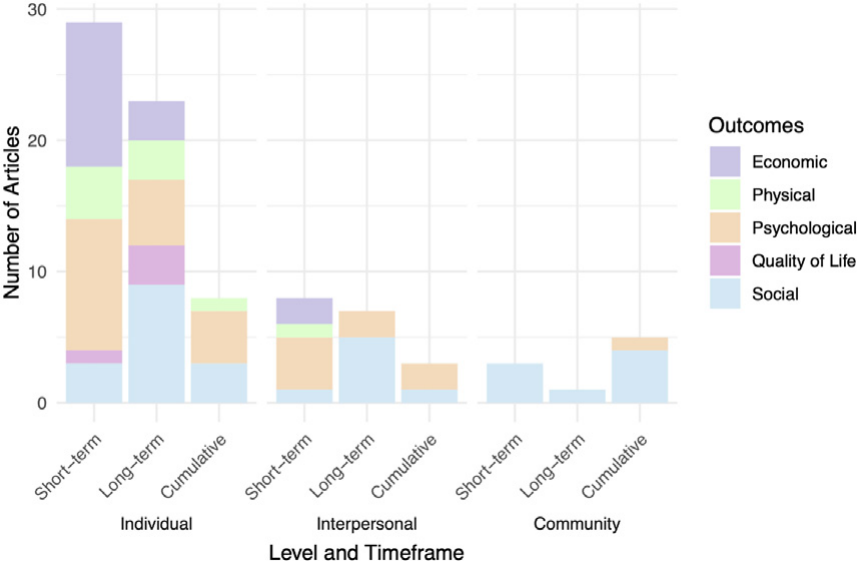
A clustered and stacked bar chart depicting the distribution of outcomes across ecological level and time.

In addition to stratification by timeframe, we stratified the findings by ecological level at which the research was conducted. This underscores a notable scarcity of research examining the impacts of firearm violence at the community level (n = 9; 10.2%), with the majority (n = 60; 69%) of included studies focusing primarily on individual-level outcomes (Fig. 3).

At the individual level, twenty-nine of the sixty studies (48.3%) evaluated short-term impacts, twenty-three (38.3%) explored long-term burdens, and eight (13.3%) highlighted cumulative harms. Most individual level studies centered on psychological outcomes, exploring direct survivor-witnesses' experiences of anxiety, depression, and post-traumatic stress disorder (PTSD) (n = 19; 67.9%). Conversely, only nine studies (10.2%) form the community level with three studies (33.3%) highlighting short-term impacts, one (11.1%) underscoring long-term outcomes, and five (55.6%) exploring cumulative effects. Most community-level studies (n = 8; 88.9%) were explorations of social impacts, highlighting how repeated exposure to violence amplifies trauma over time, leading to deeper and more intractable harms.

Adopting aspects from both the ENTREQ and SWiM guidelines, we describe the physical, psychological, economic, and social outcomes of firearm violence exposure documented within the 87 included articles below.

### Impacts on physical well-being

A total of nine studies (10.3%) explored the physical outcomes of firearm injuries, with consideration of short-term (n = 5; 55.6%), long-term (n = 3; 33.3%), and cumulative (n = 1; 11.1%) effects (Fig. 2). Eight of these articles (88.9%) reported on individual-level (i.e., survivor-witnesses) experiences, highlighting chronic complications and functional impairments (Fig. 3). Czaja and colleagues reported a retrospective case series of nonfatal injuries, including fractures, soft tissue damage, and traumatic brain injuries, sustained during mass shootings.^21^ Retained bullet fragments in patients with gunshot wounds were found to contribute to long-term complications, such as chronic pain, infection, and, in rare cases, lead toxicity.^22^^,^^23^ Zeineddin and colleagues explored the disfiguring effects of firearm injuries in children, underscoring severe, lifelong consequences for those who often require multiple reconstructive surgeries.^24^ Only one study reported on physical outcomes for children in homicide-suicide cases, identifying a range of traumatic injuries, many of which caused severe disability or death (Sillito & Salari, 2011).^25^

Of the nine studies, all but the mixed methods systematic review by Apte and colleagues utilized quantitative methods.^23^ Most (n = 5; 55.6%) were retrospective cohort studies, followed by cross-sectional studies (n = 2; 22.2%). While most studies have strong methodological foundations, those with smaller or more specific samples, such as Sillito and Salari, may face limitations in broad applicability.^25^ The effect direction across studies on physical well-being trends toward negative impacts on health and well-being, with notable subgroup effects on children and adolescents, particularly those in low-income, minoritized communities.^24^

### Attention to the psychological outcomes

Twenty-eight studies (32.2%) focused on psychological outcomes associated with firearm violence, emphasizing the profound and often long-lasting mental health consequences for both survivors and those indirectly exposed. A majority (67.9%, n = 19) of the studies explored individual-level experiences, while a smaller subset (28.6%, n = 8) investigated interpersonal outcomes, and only one (3.6%) accounted for the community-level impacts. Across both quantitative and qualitative research, studies highlighted the role of support systems and interventions in mitigating psychological distress.^26^^,^^27^

Quantitative research consistently demonstrated a strong association between exposure to firearm violence and heightened rates of PTSD, anxiety, and depression. Structured interventions such as cognitive behavioral therapy (CBT), group therapy, and community-based support programs, particularly critical for marginalized individuals with limited access to mental health care, were found to be effective in reducing psychological distress.^28^^,^^29^ However, qualitative studies (35.7%, n = 10) added depth to these findings by capturing survivors’ lived experiences, illustrating how trauma manifests in daily life, and revealing barriers to accessing mental health support.^30^ For instance, as noted in Box 1, while statistical models indicate the efficacy of CBT in symptom reduction, qualitative accounts suggest that cultural stigma, financial barriers, and distrust in the healthcare system often prevent survivors from seeking treatment, limiting generalizability of intervention outcomes.^31^, ^32^, ^33^, ^34^, ^35^, ^36^

Moreover, qualitative research identified protective factors, such as social support and personal agency, that contribute to psychological resilience and recovery.^30^ Survivor-witnesses expressed how strong family ties, peer networks, and a sense of purpose helped mitigate the long-term psychological damage caused by firearm violence. These narratives align with quantitative findings that suggest a possible positive effect direction, where increased social support correlates with improved outcomes.^28^^,^^30^ Together, these studies demonstrate that while firearm violence is strongly linked to psychological distress, the pathways to recovery are complex, shaped by both structural interventions and personal experiences.

### Quality of life

Four studies (4.6%), all (100%) at the individual level and primarily (75%) assessing long-term effects, addressed quality of life (QoL) outcomes of firearm violence. Quantitative findings consistently revealed significant reductions in both physical and mental health-related QoL among survivors, particularly those with severe injuries or complications. Both Orlas and colleagues and Herrera-Escobar and colleagues found persistent reports of physical disability and chronic pain, particularly among those with severe injuries or complications.^3^^,^^6^

In a prospective cohort study, Vella and colleagues illustrated the multi-dimensional impact of firearm injuries on mental and social well-being, noting an array of functional, psychological, and emotional outcomes including not only physical limitations but also high rates of depression, anxiety, and social withdrawal among survivors. Survivor-witness self-reports provide deeper insight into the lived reality of these effects, describing struggles with reintegration into work and social life,^37^ ongoing fears related to the shooting,^38^ and challenges in securing adequate medical and psychological support (Box 1). Qualitative narratives contextualize the quantitative data, illustrating how reduced QoL is not merely a clinical outcome but an experience shaped by social and emotional factors.

Most studies reporting on QoL (n = 3, 75%) used a cohort study design, with two (50%) incorporating prospective approaches that improve data quality by reducing recall bias and collecting real-time data on QoL changes. While the studies demonstrate robust design and careful follow-up, variations in sample size and study scope influence the generalizability and applicability of findings across different survivor populations. Nonetheless, the effect direction across these studies consistently shows ongoing, negative impacts on survivors of firearm injuries, not only through physical disability but also in psychological and social burdens.

### Social burden of firearm exposure

Thirty (34.5%) studies examined the intersection of social factors, such as family dynamics, peer relationships, and community environments, with firearm violence and its aftermath. These studies explored individual (50%, n = 15), interpersonal (23.3%, n = 7), and community-level (26.7%, n = 8) outcomes. Quantitative analyses revealed that exposure to firearm violence significantly disrupts social functioning. For example, Mitchell and colleagues found that youth who witnessed firearm violence reported heightened fear, diminished trust in their communities, and withdrawal from social activities.^39^ Nehra and colleagues identified a significant drop in firearm weapon carriage at 3- and 6-months post-injury, followed by a return to pre-injury levels at 12-months, suggesting complex behavioral adaptations to trauma.^40^

Qualitative research provided deeper insight into these trends, contextualizing social ramifications of firearm violence. As noted in Box 1, ethnographic studies of Black male survivors highlighted how cultural expectations, particularly perceptions of manhood and masculinity, shaped social recovery.^41^ Similarly, researchers found that social marginalization and trauma, reinforcing cycles of exclusion and distrust, were common outcomes in ethnic minority communities affected by firearm violence.^36^^,^^42^ These narratives complement quantitative findings on social withdrawal by revealing underlying emotional and cultural factors that shape how survivors navigate the aftermath of violence.

Combined qualitative and quantitative findings indicate a predominantly negative effect direction regarding social outcomes, including increased social isolation and community division after incidents of firearm violence. However, research also points to mitigating factors: Vicary and Fraley demonstrated that access to support networks — both online and in-person — can significantly buffer the psychological and social impacts of violence.^43^ This aligns with broader quantitative trends showing that strong social support is linked to improved mental health outcomes post-trauma. Thus, while social consequences of firearm violence are largely detrimental, findings suggest that fostering community support may mitigate these effects.

### The economic cost alongside healthcare need and utilization

Sixteen studies (18.4%) addressed the economic burden associated with firearm injuries, highlighting both direct healthcare burden and indirect societal costs. Fourteen of these (87.5%) report on individual-level (i.e., direct survivor-witness) impacts. National analyses of healthcare costs related to pediatric firearm injuries and family care demonstrated a significant rise in healthcare charges over the last two decades.^44^, ^45^, ^46^ Quantitative research further illustrated the increasing strain on healthcare systems, particularly following mass shootings, driven by survivors’ need for rapid transport services, often multiple specialized procedures, and prolonged hospital stays.^47^ Zuo and colleagues investigated sex differences in post-injury healthcare spending, revealing that male survivors tend to incur higher costs and risk readmission due to more severe injuries.^48^ Additionally, researchers found that recurrent firearm injuries were particularly common in urban trauma centers, predominantly affecting uninsured (50%), Black (96%), and male (93%) communities.^49^ Significant regional variation in costs further underscored disparities in access to care and financial strain.^50^

While quantitative studies established clear patterns in healthcare spending and financial hardship, qualitative findings illuminated the experiences behind these statistics. For instance, as noted in Box 1, survivors reported struggling with loss of employment and the cascading economic effects of long-term disability, highlighting the limitations of insurance coverage and social safety nets.^33^^,^^36^ These narratives contextualize large-scale economic trends, revealing impacts on long-term socioeconomic stability.

Of the included studies, twelve (75%) used a cohort study design, with nine (56.2%) employing a retrospective approach. Researchers increased external validity by including multiple trauma centers and diverse patient populations. However, some studies, like Zuo and colleagues, focused on more limited datasets or specific demographic groups, potentially introducing bias or limiting the generalizability of their findings.^48^ Overall, findings across methodologies consistently pointed to increased healthcare costs and financial instability among survivors. The integration of quantitative cost analyses with qualitative accounts of survivor hardship underscores the need for comprehensive interventions — both policy-driven and community-based — to mitigate the long-term economic toll of firearm exposure.

### Cross-domain insights: the interconnected burdens of firearm violence

Although we categorized findings into distinct outcome domains (psychological, physical, social, economic, and quality of life) by the focus of their primary analysis, many studies highlighted the interconnected nature of these burdens, revealing how impacts of firearm violence ripple outward across multiple domains and ecological levels. For example, psychological trauma often manifested in social withdrawal,^12^ reinforcing isolation^38^ and economic instability,^36^ while chronic physical injuries led to long-term healthcare costs and reduced quality of life.^33^ Research on support systems illustrated how strong social networks could buffer psychological distress and improve overall well-being, demonstrating that protective factors are just as multifaceted as the harms they counteract.^31^, ^32^, ^33^, ^34^, ^35^^,^^51^ By integrating qualitative narratives with quantitative patterns, this review highlights the necessity of cross-sectoral interventions that acknowledge the complex, compounding nature of firearm violence’s consequences.

## Discussion

To our knowledge, this is the first scoping review that encompasses 25 years of literature to explore the ecological landscape of firearm violence. The findings of this scoping review emphasize that research on the enduring impacts of firearm violence, especially on the interpersonal and community level, are exceptionally limited. By applying a dynamic socioecological lens, we are able to better conceptualize the multi-dimensional impacts of firearm violence as it intersects with individual, interpersonal, community-level factors.

Consistent with previous literature,^3^^,^^13^ studies in this review confirm high rates of psychological trauma, chronic pain, and long-term functional impairments among those physically injured. Our review also shows that individuals exposed to firearm violence often experience additional burdens of social isolation and stigmatization, particularly within racially marginalized communities.^36^^,^^42^ Studies on the interpersonal level (n = 18; 20.7%), highlight that exposure to firearm violence can exacerbate survivors’ sense of vulnerability and decrease their ability to engage with supportive networks shown to mitigate negative mental health outcomes and improve resilience.^26^^,^^27^ Community-level studies (n = 9; 10.4%) underscore that cumulative, or repeated, firearm violence often results in reports of heightened fear, a diminished sense of safety, and weakened social cohesion.^39^

This review highlights a range of research gaps that deserve further attention. The disproportionate focus on mass shootings (n = 23; 26.4%) and short-term psychological outcomes (n = 14; 16.1%), as opposed to cumulative, interpersonal violence (n = 3; 3.4%), limits our conceptualization of the broader spectrum of firearm violence. Few studies adequately account for indirect exposures to firearm violence, such as the relational impact of injury and trauma on families and caregivers (n = 18; 20.7%). Moreover, a significant proportion of studies (n = 46; 52.9%) did not specify the type of firearm violence or failed to disaggregate the data. This disaggregation or “splitting” is crucial, as different forms of firearm violence exposure (e.g., mass shootings vs. intimate partner violence) necessitate distinct and diverse immediate and long-term responses.^52^

While the visual summary of evidence (Fig. 2) displays the spread of existing literature, perhaps its more significant contribution lies in its blank spaces, illuminating areas for future study. The relatively empty space across interpersonal and community levels, particularly within physical, quality of life, and economic outcomes, signals that further research is needed to conceptualize the impacts of indirect trauma on the close networks of survivors as well as in communities with additional shared identities (e.g., lesbian, gay, bisexual, transgender, queer, or questioning (LGBTQ+) populations, communities of color). It is crucial to note that while there is a substantial body of research on the physical impacts of firearm violence in emergency and surgical settings, studies examining the lasting physical and quality of life consequences beyond immediate clinical interventions appear to be much more limited. This gap in the literature highlights an important area for future research. An additional frontier for research is the role of media, which may serve as a peripheral but significant source of exposure. Future studies could explore the health impacts of this broader exposure and how it may contribute to the ripple effects of firearm violence, particularly in relation to mental health outcomes, collective trauma, and societal perceptions of safety.

The key strength of this review lies in its compilation of the multifaceted impacts of firearm violence exposure, drawing on both quantitative data and qualitative narratives to provide a comprehensive view of physical, psychological, quality of life, social, and economic outcomes. However, inherent limitations exist when attempting to synthesize heterogenous and complex evidence. The team collaboratively addressed ambiguities in meetings; however, we cannot entirely rule out potential oversights. Additionally, due to resource constraints, we conducted searches only in English and excluded books. These exclusions might have led us to overlook additional insights, especially theoretical perspectives that may be discussed in book chapters. Since we did not conduct a formal quality appraisal, readers should view the general trends and effects as indicative rather than definitive. While the time frame for this review was carefully chosen to capture studies from the past 25 years, it is important to note that the period ends in March 2024. Given the growing interest and research activity in the field, particularly in the last year, there may be important studies published after our review period that are not included. However, evidence in this review indicates that the ripples of firearm violence extend across physical, psychological, social and economic spheres. Early psychosocial interventions, improved social support structures, and targeted healthcare policies may better support survivors and mitigate the broader societal impacts of firearm violence.

## Contributors

DMG and DKH contributed to the conceptualization of the review and developed the protocol, which was reviewed and approved by coauthors before registration. DMG and DKH managed the project and facilitated communication among the authors. DMG and SS the searches, screened the records, and extracted the data. All authors had access to the complete dataset presented in the manuscript and contributed to the interpretation of the data. DMG and DKH prepared the initial draft of the report. All authors (DMG, SS, DSK, SFJ, EJK, GJM, DKH) critically reviewed the draft for significant intellectual content, approved the final version for publication, and held ultimate responsibility for the decision to submit the manuscript.

## Declaration of interests

EJK held a leadership role at the Association of Out Surgeons and Allies Secretary from 2022 to 2024 and is a CeaseFirePA Board member since 2024. All remaining authors declare no competing interests.
