# Supplementary material for: Understanding the broader impacts of non-fatal firearm violence trauma in the United States: a scoping review
Source: Lancet Reg Health Am. 2025 Apr 19;46:101091. doi: 10.1016/j.lana.2025.101091 (PMC12032914; doi:10.1016/j.lana.2025.101091)
Supplement: Supplementary Fig. S1 and Table S1 [file mmc1.pdf]

Supplementary Appendix for *Understanding the broader impacts of non-fatal firearm violence trauma in the United States: A scoping review*

Deanna M. Giraldi, MPH; Susan Swingler, MPhil; David S. Kirk, PhD; Sara F. Jacoby, PhD; G.J. Melendez-Torres, DPhil; Elinore J. Kaufman, MD; David K. Humphreys, PhD

<u>**Table of Contents**</u>

Supplementary Methods: Search Strings……………………………………………………1

Supplementary Figure 1: List of Included Article Sources…………………………………3

Supplementary Table 1: Study Characteristics and Main Findings of Included Studies (1999-2024)………………………………………………………………………………………10

Supplementary Methods - Search Strings

ProQuest

1. noft(shooting* OR gun violence* OR firearm) AND noft(survivor* OR witness*) After April 1999
2. noft(shooting* OR gun violence* OR firearm) AND noft(trauma* OR consequence*) AND noft(survivor* OR witness*) After April 1999
3. noft(shooting* OR gun violence* OR firearm) AND noft(survivor* OR witness*) AND noft(burden OR cost OR harm) After April 1999

Web of Science

1. shooting* or gun violence* or firearm (Topic) and trauma or consequence or harm or witness or injury or survivor (Topic)
2. shooting* or gun violence* or firearm (Topic) and trauma or consequence or harm or witness or injury or survivor (Topic) and United States or USA or America (Topic)

PubMed & SCOPUS

1. ((shooting*) AND ((trauma*) OR (consequence*)) AND ((survivor*) OR (witness*)))
2. (((gun violence) AND (trauma*)) AND ((survivor*) OR (witness*)))

(“gun violence"[MeSH Terms] OR ("gun"[All Fields] AND "violence"[All Fields]) OR "gun violence"[All Fields]) AND "trauma"[All Fields] AND ("survivor*"[All Fields] OR "witness*"[All Fields])*

3. (((gun) OR (firearm*)) AND ((trauma*) OR (injury) OR (consequence) OR (burden) OR (cost) OR (harm)) AND ((survivor*) OR (witness*) OR (non-fatal)))

("firearms"[MeSH Terms] OR "firearms"[All Fields] OR "gun"[All Fields] OR "firearm"[All Fields]) AND ("trauma*"[All Fields] OR ("injure"[All Fields] OR "injured"[All Fields] OR "injuries"[MeSH Subheading] OR "injuries"[All Fields] OR "wounds and injuries"[MeSH Terms] OR ("wounds"[All Fields] AND "injuries"[All Fields]) OR "wounds and injuries"[All Fields] OR "injurious"[All Fields] OR "injury s" [All Fields] OR "injuryed"[All Fields] OR "injurys"[All Fields] OR "injury"[All Fields]) OR ("consequence"[All Fields] OR "consequences"[All Fields] OR "consequent"[All Fields] OR "consequently"[All Fields] OR "consequents"[All Fields]) OR ("burden"[All Fields] OR "burdened"[All Fields] OR "burdening"[All Fields] OR "burdens"[All Fields]) OR*

("economics"[MeSH Subheading] OR "economics"[All Fields] OR "cost" [All Fields] OR "costs and cost analysis"[MeSH Terms] OR ("costs"[All Fields] AND "cost"[All Fields] AND "analysis"[All Fields]) OR "costs and cost analysis"[All Fields]) OR "harm"[All Fields]) AND ("survivor"[All Fields] OR "witness*"[All Fields] OR "non-fatal"[All Fields])*

4. ((gun) AND ((trauma*) OR (consequence*)) AND ((survivor*) OR (witness*) OR (non-fatal)))

("firearms"[MeSH Terms] OR "firearms"[All Fields] OR "gun"[All Fields]) AND ("trauma"[All Fields] OR "consequence*"[All Fields]) AND ("survivor*"[All Fields] OR "witness*"[All Fields] OR "non-fatal"[All Fields])*

Supplementary Figure 1 - List of Included Article Sources

1	Andrade EG, Uberoi M, Hayes JM, Thornton M, Kramer J, Punch LJ. The impact of retained bullet fragments on outcomes in patients with gunshot wounds. *The American Journal of Surgery* 2022;**223**:787–91. doi:10.1016/j.amjsurg.2021.05.022.
2	Apte A, Bradford K, Dente C, Smith RN. Lead toxicity from retained bullet fragments: A systematic review and meta-analysis. *Journal of Trauma and Acute Care Surgery* 2019;**87**:707–16. doi:10.1097/ta.0000000000002287.
3	Ayers L. The Lived Experience of American Red Cross Disaster Mental Health Responders on MCI – Mass Shooting Deployments. ProQuest Dissertations and Theses Global 2019.
4	Baker NS, VanHook C, Ricks T, Vil CSt, Lassiter T, Bonne S. Protect and provide: Perceptions of manhood and masculinities among disabled violently injured black men in a hospital-based violence intervention program. *American Journal of Men's Health* 2024;**18**. doi:10.1177/15579883231221390.
5	Barbato CA. "Embracing their memories": Accounts of loss and May 4, 1970. *Journal of Loss and Trauma* 2003;**8**:73–98. doi:10.1080/15325020305867.
6	Beardslee J, Mulvey E, Schubert C, Allison P, Infante A, Pardini D. Gun- and Non-Gun–related violence exposure and risk for subsequent gun carrying among male juvenile offenders. *Journal of the American Academy of Child & Adolescent Psychiatry* 2018;**57**:274–9. doi:10.1016/j.jaac.2018.01.012.
7	Bongiorno DM, Badolato GM, Boyle M, Vernick JS, Levy JF, Goyal MK. United States trends in healthcare charges for pediatric firearm injuries. *The American Journal of Emergency Medicine* 2021;**47**:58–65. doi:10.1016/j.ajem.2021.03.046.
8	Borg BA, Rabinak CA, Marusak HA. Violence exposure and mental health consequences among urban youth. *Current Psychology* 2021;**42**:8176–85. doi:10.1007/s12144-021-02141-4.
9	Boschert EN, Stubblefield CE, Reid KJ, Schwend RM. Twenty-two years of pediatric musculoskeletal firearm injuries: Adverse outcomes for the very young. *Journal of Pediatric Orthopaedics* 2020;**41**. doi:10.1097/bpo.0000000000001682.
10	Bruns A. Posttraumatic Growth and Spiritual Well-Being in Survivors of the Columbine High School Shooting. Regent University Doctoral Thesis. 2013.
11	Comer BP, Connolly EJ. Exposure to gun violence and handgun carrying from adolescence to adulthood. *Social Science & Medicine* 2023;**328**:115984. doi:10.1016/j.socscimed.2023.115984.
12	Czaja M, Kraus C, Phyo S, Olivieri P, Mederos D, Mohammed S, et al. Nonfatal injuries sustained in mass shootings in the US, 2012-2019: Injury diagnosis matrix, incident context, and public health considerations. *Western Journal of Emergency Medicine* 2023;**24**. doi:10.5811/westjem.58395.
13	Deng H, Yue JK, Winkler EA, Dhall SS, Manley GT, Tarapore PE. Pediatric firearm-related traumatic brain injury in United States Trauma Centers. *Journal of Neurosurgery: Pediatrics* 2019;**24**:498–508. doi:10.3171/2019.5.peds19119.
14	Douglas A, Rogers S. Survivor's remorse. Journal of Trauma and Acute Care Surgery 2023. doi:10.1097/ta.0000000000004175.
15	Felix ED, Dowdy E, Green JG. University student voices on healing and recovery following tragedy. *Psychological Trauma: Theory, Research, Practice, and Policy* 2018;**10**:76–86. doi:10.1037/tra0000172.

16	Fergus TA, Rabenhorst MM, Orcutt HK, Valentiner DP. Reactions to trauma research among women recently exposed to a campus shooting. Journal of Traumatic Stress 2011;**24**:596–600. doi:10.1002/jts.20682.
17	Greenspan AI, Kellermann AL. Physical and psychological outcomes 8 months after serious gunshot injury. *The Journal of Trauma: Injury, Infection, and Critical Care* 2002;**53**:709–16. doi:10.1097/00005373-200210000-00015.
18	Grills-Taquechel AE, Littleton HL, Axsom D. Social Support, world assumptions, and exposure as predictors of anxiety and quality of life following a mass trauma. *Journal of Anxiety Disorders* 2011;**25**:498–506. doi:10.1016/j.janxdis.2010.12.003.
19	Hawkins NA, McIntosh DN, Silver RC, Holman EA. Early responses to school violence. *Journal of Emotional Abuse* 2007;**4**:197–223. doi:10.1300/j135v04n03_12.
20	Herrera-Escobar JP, Rivero R, Apoj M, Geada A, Villanyi M, Blake D, et al. Long-term social dysfunction after trauma: What is the prevalence, risk factors, and associated outcomes? *Surgery* 2019;**166**:392–7. doi:10.1016/j.surg.2019.04.004.
21	Hink AB, Atkins DL, Rowhani-Rahbar A. Not all survivors are the same: Qualitative assessment of prior violence, risks, recovery and perceptions of firearms and violence among victims of Firearm Injury. *Journal of Interpersonal Violence* 2021;**37**. doi:10.1177/08862605211005157.
22	Holloway K, Cahill G, Tieu T, Njoroge W. Reviewing the literature on the impact of gun violence on early childhood development. *Current Psychiatry Reports* 2023;**25**:273–81. doi:10.1007/s11920-023-01428-6.
23	Hootman JM, Annest JL, Mercy JA, Ryan GW, Hargarten SW. National estimates of non-fatal firearm related injuries other than gunshot wounds. *Injury Prevention* 2000;**6**:268–74. doi:10.1136/ip.6.4.268.
24	Hureau DM, Wilson T, Rivera-Cuadrado W, Papachristos AV. The experience of secondary traumatic stress among Community Violence Interventionists in Chicago. *Preventive Medicine* 2022;**165**:107186. doi:10.1016/j.ypmed.2022.107186.
25	Johnson SD, North CS, Smith EM. Psychiatric Disorders Among Victims of a Courthouse Shooting Spree: A Three-Year Follow-Up Study. *Community Mental Health Journal* 2002;**38**:181–94. doi:10.1023/a:1015269521969.
26	Jones KA. A seat at the table: Centering the voices of gun violence survivors. *Pediatrics* 2020;**146**. doi:10.1542/peds.2020-015974.
27	Joseph B, Hanna K, Callcut RA, Coleman JJ, Sakran JV, Neumayer LA. The hidden burden of mental health outcomes following firearm-related injuries. *Annals of Surgery* 2019;**270**:593–601. doi:10.1097/sla.0000000000003473.
28	King C, Huang X. Neighbourhood violence and housing instability: An exploratory study of low-income women. *Housing Studies* 2022;**39**:651–70. doi:10.1080/02673037.2022.2074970.
29	Kwon EG, Wang BK, Iverson KR, O’Connell KM, Nehra D, Rice-Townsend SE. Interpersonal violence affecting the pediatric population: Patterns of injury and recidivism. *Journal of Pediatric Surgery* 2023;**58**:136–41. doi:10.1016/j.jpedsurg.2022.09.009.
30	Lal A, John KJ, Mishra AK, Sherif AA. Burden of firearm-related injuries as associated secondary diagnosis in the United States from National Inpatient Sample Dataset. *Internal and Emergency Medicine* 2023;**18**:457–65. doi:10.1007/s11739-022-03190-8.

31	Leibbrand C, Rivara F, Rowhani-Rahbar A. Gun violence exposure and experiences of depression among mothers. *Prevention Science* 2021;**22**:523–33. doi:10.1007/s11121-020-01202-7.
32	Lennon T, Kemal S, Heffernan ME, Bendelow A, Sheehan K, Davis MM, et al. Childhood exposure to firearm violence in Chicago and its impact on Mental Health. *Academic Pediatrics* 2024;**24**:982–6. doi:10.1016/j.acap.2023.12.001.
33	Liu SR, Kia-Keating M. A novel examination of exposure patterns and posttraumatic stress after a university mass murder. *Psychological Trauma: Theory, Research, Practice, and Policy* 2019;**11**:176–83. doi:10.1037/tra0000354.
34	Lumbard DC, Nygaard RM, Richardson CJ, Liao LF, Stewart RM, Eastridge BJ, et al. Burden of unintentional pediatric firearm injury: An examination of the nationwide readmission database. *Journal of Trauma and Acute Care Surgery* 2023;**95**:419–25. doi:10.1097/ta.0000000000003930.
35	Magee LA, Aalsma MC, Fortenberry JD, Gharbi S, Wiehe SE. Mental health outcomes from direct and indirect exposure to firearm violence: A cohort study of nonfatal shooting survivors and family members. *Journal of Criminal Justice* 2022;**83**:101961. doi:10.1016/j.jcrimjus.2022.101961.
36	Magee LA, Ortiz D, Adams ZW, Marriott BR, Beverly AW, Beverly B, et al. Engagement with mental health services among survivors of Firearm Injury. *JAMA Network Open* 2023;**6**. doi:10.1001/jamanetworkopen.2023.40246.
37	Mancini AD, Littleton HL, Grills AE. Can people benefit from acute stress? social support, psychological improvement, and resilience after the virginia tech campus shootings. *Clinical Psychological Scienc*e 2015;**4**:401–17. doi:10.1177/2167702615601001.
38	McCall WT. Caring for patients from a school shooting: A qualitative case series in emergency nursing. *Journal of Emergency Nursing* 2020;**46**. doi:10.1016/j.jen.2020.06.005.
39	McGee ZT, Logan K, Samuel J, Nunn T. A multivariate analysis of gun violence among urban youth: The impact of direct victimization, indirect victimization, and victimization among peers. *Cogent Social Sciences* 2017;**3**:1328772. doi:10.1080/23311886.2017.1328772.
40	McLaughlin K, Kar JA. Aftermath of the parkland shooting: A case report of post-traumatic stress disorder in an adolescent survivor. *Cureus 2019*. doi:10.7759/cureus.6146.
41	Mitchell KJ, Jones LM, Turner HA, Beseler CL, Hamby S, Wade R. Understanding the impact of seeing gun violence and hearing gunshots in public places: Findings from the Youth Firearm Risk and safety study. Journal of Interpersonal Violence 2019;**36**:8835–51. doi:10.1177/0886260519853393.
42	Molina O, Jacinto G, & Yegidis B. The Pulse Nightclub Mass Shooting, and Factors Affecting Community Resilience Following the Terrorist Attack. *Best Practices in Mental Health* 2019;**15**:2.
43	Molnar BE, Miller MJ, Azrael D, Buka SL. Neighborhood predictors of concealed firearm carrying among children and adolescents. Archives of Pediatrics & Adolescent Medicine 2004;**158**:657. doi:10.1001/archpedi.158.7.657.
44	Mooney EJ. The Effects of Witnessing a School Shooting on a Teacher's Career. Dissertation 2013. *https://doi.org/10.17760/d20003339*

45	Mueller KL, Cooper BP, Moran V, Lew D, Ancona R, Landman JM, et al. Incidence of and factors associated with recurrent firearm injury among patients presenting to St. Louis Trauma Centers, 2010 to 2019. *Annals of Internal Medicine* 2023;**176**:1163–71. doi:10.7326/m23-0069.
46	Nehra D, Bulger EM, Maier RV, Moloney KE, Russo J, Wang J, et al. A prospective US National Trauma Center Study of firearm injury survivors weapon carriage and posttraumatic stress disorder symptoms. *Annals of Surgery* 2021;**274**. doi:10.1097/sla.0000000000005043.
47	North CS. Three-year follow-up of survivors of a mass shooting episode. *Journal of Urban Health: Bulletin of the New York Academy of Medicine* 2002;**79**:383–91. doi:10.1093/jurban/79.3.383.
48	O'Neill KM, Vega C, Saint-Hilaire S, Jahad L, Violano P, Rosenthal MS, et al. Survivors of gun violence and the experience of recovery. *Journal of Trauma and Acute Care Surgery* 2020;**89**:29–35. doi:10.1097/ta.0000000000002635.
49	O'Neill KM, Jean RA, Dodington J, Davis K, Becher RD. Evaluation of firearm-related Reinjury in Connecticut: An opportunity for gun violence prevention. *Journal of Surgical Research* 2022;**274**:23–30. doi:10.1016/j.jss.2021.12.009.
50	Oddo ER, Maldonado L, Hink AB, Simpson AN, Andrews AL. Increase in mental health diagnoses among youth with nonfatal firearm injuries. *Academic Pediatrics* 2021;**21**:1203–8. doi:10.1016/j.acap.2021.06.003.
51	Orlas CP, Thomas A, Herrera-Escobar JP, Price MA, Haider AH, Bulger EM. Long-term outcomes of firearm injury survivors in the United States. *Annals of Surgery* 2021;**274**:962–70. doi:10.1097/sla.0000000000005204.
52	Phillips R, Shahi N, Bensard D, Meier M, Shirek G, Goldsmith A, et al. Guns, scalpels, and sutures: The cost of gunshot wounds in children and adolescents. *Journal of Trauma and Acute Care Surgery* 2020;**89**:558–64. doi:10.1097/ta.0000000000002766.
53	Piskorik JM. A narrative inquiry into the influence of school shooting survival on college transition and experience. The Research Repository @ West Virginia University. 2020. https://researchrepository.wvu.edu/etd/11616
54	Pulcini CD, Goyal MK, Hall M, De Souza HG, Chaudhary S, Alpern ER, et al. Mental health utilization and expenditures for children pre–post firearm injury. *American Journal of Preventive Medicine* 2021;**61**:133–5. doi:10.1016/j.amepre.2021.01.024.
55	Richardson JB, Wical W, Kottage N, Bullock C. Shook ones: Understanding the intersection of nonfatal violent firearm injury, incarceration, and traumatic stress among young black men. *American Journal of Men's Health* 2020;**14**. doi:10.1177/1557988320982181.
56	Richmond TS, Lemaire J. Years of life lost because of gunshot injury to the brain and spinal cord. American Journal of Physical Medicine & Rehabilitation 2008;**87**:609–18. doi:10.1097/phm.0b013e31817fb496.
57	Rivara FP, Hink AB, Kuhls D, Banks S, Agoubi LL, Kirkendoll S, et al. Firearm injuries in Missouri. *PLOS ONE* 2023;**18**. doi:10.1371/journal.pone.0294737.

58	Rosas S, Gwam CU, Araiza ET, Roche MW, Emory CL, Carroll EA, et al. Economic impact of orthopaedic care for non-fatal gunshot wounds: Analysis of a public health crisis. *Annals of Translational Medicine* 2021;**9**:210–210. doi:10.21037/atm-20-1064.
59	Rowhani-Rahbar A, Zatzick D, Wang J, Mills BM, Simonetti JA, Fan MD, et al. Firearm-related hospitalization and risk for subsequent violent injury, death, or crime perpetration. *Annals of Internal Medicine* 2015;**162**:492–500. doi:10.7326/m14-2362.
60	Ryan J, Hawdon J. From individual to community: The “framing” of 4-16 and the display of Social Solidarity. *Traumatology* 2008;**14**:43–51. doi:10.1177/1534765607312686.
61	Sabri B, Campbell JC. Firearm-related risks and consequences for immigrant women in abusive relationships: Barriers to reporting threats to safety and recommendations for Safety Planning. *Journal of Aggression, Maltreatment & Trauma* 2023;**33**:407–31. doi:10.1080/10926771.2023.2224257.
62	Sarani B, Smith ER, Shapiro G, Nahmias J, Rivas L, McIntyre R, et al. Characteristics of survivors of civilian public mass shootings: An Eastern Association for the surgery of trauma multicenter study. *Journal of Trauma and Acute Care Surgery* 2021;**90**:652–8. doi:10.1097/ta.0000000000003069.
63	Semenza DC, Stansfield R. Non-fatal gun violence and Community Health Behaviors: A neighborhood analysis in Philadelphia. Journal of Behavioral Medicine 2021;**44**:833–41. doi:10.1007/s10865-021-00232-y.
64	Semenza DC, Stansfield R. Community gun violence and functional disability: An ecological analysis among men in four U.S. cities. *Health & Place* 2021;**70**:102625. doi:10.1016/j.healthplace.2021.102625.
65	Semenza DC, Daruwala S, Brooks Stephens JR, Anestis MD. Gun violence exposure and suicide among Black Adults. *JAMA Network Open* 2024;**7**. doi:10.1001/jamanetworkopen.2023.54953.
66	Shockley AG. Co-Victims of Gun Violence: How Black Women Navigate Spaces of Trauma. *Temple University Dissertations* 2023.
67	Sillito CL, Salari S. Child outcomes and risk factors in U.S. homicide-suicide cases 1999–2004. *Journal of Family Violence* 2011;**26**:285–97. doi:10.1007/s10896-011-9364-6.
68	Smith RN, Seamon MJ, Kumar V, Robinson A, Shults J, Reilly PM, et al. Lasting impression of violence: Retained bullets and depressive symptoms. *Injury* 2018;**49**:135–40. doi:10.1016/j.injury.2017.08.057.
69	Smith CP, Cheatham ML, Safcsak K, Emrani H, Ibrahim JA, Gregg M, et al. Injury characteristics of the pulse nightclub shooting: Lessons for Mass Casualty Incident Preparation. *Journal of Trauma and Acute Care Surgery* 2019;**88**:372–8. doi:10.1097/ta.0000000000002574.
70	Smith RN, Nedergaard RM, Meyer CH, DeSousa N, Ghosh M, Blount Q, et al. Psychological impacts of retained bullets from the perspective of survivors. *The American SurgeonTM* 2023;**89**:3732–8. doi:10.1177/00031348231171121.
71	Smith R. Virginia Secondary-School Counselors’ Descriptions of Student Reactions to National Mass Shootings. Dissertation 2020.

72	Song Z, Zubizarreta JR, Giuriato M, Paulos E, Koh KA. Changes in health care spending, use, and clinical outcomes after nonfatal firearm injuries among survivors and family members. *Annals of Internal Medicine* 2022;**175**:795–803. doi:10.7326/m21-2812.
73	Song Z, Zubizarreta JR, Giuriato M, Koh KA, Sacks CA. Firearm injuries in children and adolescents: Health and economic consequences among survivors and family members. *Health Affairs* 2023;**42**:1541–50. doi:10.1377/hlthaff.2023.00587.
74	Spearman KJ, Marineau L, Owolabi A, Alexander KA, Campbell J. Firearms and post-separation abuse: Providing context behind the data on firearms and intimate partner violence. *Journal of Advanced Nursing* 2023;**80**:1484–96. doi:10.1111/jan.15933.
75	Spinuzzi A. Mass Shootings and Trauma: A Qualitative Study on How Survivor Perception Affects Trauma Response. The Chicago School of Professional Psychology ProQuest Dissertations & Theses 2022. 29325083.
76	Spitzer SA, Forrester JD, Tennakoon L, Spain DA, Weiser TG. A decade of hospital costs for firearm injuries in the United States by region, 2005–2015: Government Healthcare costs and firearm policies. *Trauma Surgery & Acute Care Open* 2022;**7**. doi:10.1136/tsaco-2021-000854.
77	Turner HA, Mitchell KJ, Jones LM, Hamby S, Wade R, Beseler CL. Gun violence exposure and posttraumatic symptoms among children and Youth. *Journal of Traumatic Stress* 2019;**32**:881–9. doi:10.1002/jts.22466.
78	Vella MA, Warshauer A, Tortorello G, Fernandez-Moure J, Giacolone J, Chen B, et al. Long-term functional, psychological, emotional, and social outcomes in survivors of firearm injuries. *JAMA Surgery* 2020;**155**:51. doi:10.1001/jamasurg.2019.4533.
79	Vicary AM, Fraley RC. Student reactions to the shootings at Virginia Tech and Northern Illinois University: Does sharing grief and support over the internet affect recovery? *Personality and Social Psychology Bulletin* 2010;**36**:1555–63. doi:10.1177/0146167210384880.
80	Vinall DJ. Massacre Survivor Syndrome: Trauma Sequelae and Life Adjustment in Survivors of Mass Shootings Treated with EMDR. Dissertation 2020.
81	Wallace JG, Chernet R, Formica MK, Adeonigbagbe O, Flores RL, Marchesani R, et al. Gun violence and the voices of youth on Community Safety in the time of covid-19 in East Harlem, NY: A youth participatory action research cross-sectional study. *Injury Epidemiology* 2023;**10**. doi:10.1186/s40621-023-00440-x.
82	Webb PB, Jimenez J, Elder A, Cotte AO, Ravichandran A, Holbrook E, et al. Exploring lived experiences of gunshot wound survivors: A key to ethnographically informed public health interventions for curbing firearm violence. *Injury* 2024;**55**:111240. doi:10.1016/j.injury.2023.111240.
83	Weigend Vargas E, Hemenway D. Emotional and physical symptoms after gun victimization in the United States, 2009–2019. *Preventive Medicine* 2021;**143**:106374. doi:10.1016/j.ypmed.2020.106374.
84	Wintemute GJ, Aubel AJ, Pallin R, Schleimer JP, Kravitz-Wirtz N. Experiences of violence in daily life among adults in California: A population-representative survey. *Injury Epidemiology* 2022;**9**. doi:10.1186/s40621-021-00367-1.

85	Zeineddin S, Zeineddin A, Jain A, Olufajo OA, Koolaee S, Cornwell EE, et al. Disfiguring firearm injuries in children in the United States. *The American Surgeon^{TM}* 2021;**89**:2070–2. doi:10.1177/00031348211023432.
86	Zhao P. Minor feelings in the wake of the Atlanta Attack: How a mom of Asian descent spent the first 100 hours in the aftermath. *Cultural Studies ↔ Critical Methodologies* 2021;**21**:351–4. doi:10.1177/15327086211019954.
87	Zuo Y, Pino EC, Vyliparambil M, Kalesan B. Sex differences in early cardiovascular and all-cause hospitalization outcomes after surviving firearm injury. *American Journal of Men's Health* 2018;**12**:1029–38. doi:10.1177/1557988318761989.

Supplementary Table 1 - Study Characteristics and Main Findings of Included Studies (1999-2024)

Article	Aim of study	Study design	Main Findings
**Andrade et al., 2022[1]**	To explore the impact of retained bullet fragments (RBF) in victims of non-fatal firearm injuries	Retrospective Cohort Study	RBFs may represent an unrecognized risk factor for both repeat ED visits and subsequent bullet injury. Patients with RBFs were more likely admited from ED (57.4% v. 41.8%, p <0.001) and had higher rates of return to ED in 6 months (30.7% vs 18.6%, p<0.001) and higher rates of subsequent GSW in the next year (5.1% vs 1.8%, p=0.03).
**Apte et al., 2019[2]**	To determine whether there is an association among retained bullet fragments (RBF), elevated blood lead levels (BLL) and lead toxicity in survivors of firearm injury 16 years and older	Systematic Review (Mixed Methods)	Patients with bony fractures or multiple RBF, who are at higher risk of elevated BLL, should be monitored for BLL in intervals of 3 months within the first year of injury. For patients who return with BLL above 5 μg/dL, all efforts must be undertaken to remove fragments if there is no potential to worsen the injury.
**Ayers, 2020[3]**	To listen, describe, interpret, and make meaning of the lived experiences of American Red Cross (ARC) Disaster Mental Health (DMH) responders who were deployed to Mass Casualty Incidents (MCIs), specifically mass shootings, between 2008-2018	Phenomenological (Qualitative Dissertation)	DMH responders are both negatively and positively affected by their work on human caused intentional MCIs, specifically mass shootings. The study revealed that responders who work with grief and trauma populations appear to have the professional skills and experience which assist them in successfully processing client traumatic material
**Baker et al., 2024[4]**	To explore how Black men who suffer from disabilities via a firearm negotiated their masculine identities	Ethnography (Qualitative)	Three themes emerged: (1) perceptions of manhood, (2) loss of independence and burden on others, and (3) and mobility. These themes highlighted and described how their lives were impacted post-injury and characterized their psychological and physical experience of recovery. Black men continue to be hyper-invisible when discussing violently acquired disabilities.
**Barbato, 2003[5]**	To elevate voices of survivors and eye-witnesses from May 4, 1970 Kent State University shooting	Case Studies (Qualitative)	Part of the healing process in dealing with a traumatic loss is being able to publicly tell the stories of the loss of our loved ones and publicly and privately "embracing their memories"
**Beardslee et al.,2018[6]**	To determine whether juvenile offenders exhibit an increased propensity to carry a firearm after being exposed to gun violence and/or non-gun violence	Retrospective Cohort Study (Quantitative)	The study indicates that young men with a history of criminal offending are more likely to carry a gun after being exposed to gun-related violence in the community. There was no evidence that exposure to non-gun violence conferred the same risk for future gun carrying.
**Bongiorno et al., 2021[7]**	To estimate mean charges for initial ED and inpatient care for acute firearm injuries among children in the U.S., compare differences in charges by firearm injury intent among children, and evaluate trends in charges for pediatric firearm injuries over time, including within intent subgroups	Cross Sectional Study (Quantitative)	Injuries were 53.9% assault-related, 37.7% unintentional, 1.8% self-inflicted, and 6.7% undetermined. Self-inflicted injuries had higher mean charges ($98,988) than assault-related ($52,496) and unintentional ($28,618) injuries (p < 0.001). Self-inflicted injuries remained associated with higher mean charges relative to unintentional injuries, after adjusting for patient demographics, hospital characteristics, and injury severity (p = 0.015). Mean charges for pediatric firearm-related injuries increased over time (p-trend = 0.018) and were 23.1% higher in 2016 versus 2009.
**Borg et al., 2023[8]**	To examine the prevalence of violence exposure and associated mental health consequences among urban and non-urban youth	Cross-Sectional Study	Urban youth reported greater exposure to gun violence than their non-urban counterparts. They also reported significantly higher depressive symptoms and greater violence exposure was associated with higher anxiety symptoms among urban youth. Individual exposures, particularly hearing gunshots, were significant predictors of anxiety and depressive symptoms.
**Boschert et al., 2021[9]**	To investigate the 22-year experience of pediatric firearm-related musculoskeletal injuries at a major pediatric level 1 hospital and analyze the risk of adverse outcomes in children under 10 years of age	Retrospective Cohort Study (Quantitative)	Over one fourth of survivors of musculoskeletal firearm injuries had an adverse outcome (death, growth disturbance, amputation, impairment). Children 10 years and above represent the majority of firearm injuries in our population; however, when injured, those below 10 years are more likely to have an adverse outcome.
**Bruns, 2014[10]**	To examine the relationship between posttraumatic growth (PTG) and spiritual well-being (SWB) in the survivors of the Columbine High School shooting	Correlational Research (Quantitative Dissertation)	A statistically significant relationship exists between gender and PTG and SWB; there is a statistically significant relationship between proximity to the shooting and PTG; PTG and relationship with those who were injured or killed was not a statistically significant finding; as PTG increases SWB increases as well as the inverses
**Comer & Connolly, 2023[11]**	To evaluate the relations between witnessing gun violence before age 12 and subsequent handgun-carrying behavior from adolescence to adulthood	Retrospective Cohort Study (Quantitative)	Childhood exposure to gun violence appears to be a risk factor for handgun carrying in adolescence. Nonetheless, other behaviors and demographic characteristics account for inter-individual differences in changes in handgun carrying across the life course.
**Czaja et al., 2023[12]**	To explore non-fatal outcomes of victims of mass shootings	Retrospective Case Series (Quantitative)	Survivors of mass shootings have substantial morbidity and characteristic injury distribution, but 37% of victims had no GSW.
**Deng et al., 2019[13]**	To systematically characterize the epidemiology of and outcomes following gunshot wounds to the head in a pediatric population-based sample to identify predictors of prolonged hospitalization, morbidity, and death	Retrospective Cohort Study (Quantitative)	Assault (63.0%), handguns (45.6%), and injury incurred in a residential area (40.6%) were most common. The mean hospital length of stay was 11.6 ± 14.4 days for the survivors. Mortality was 56.5% with injury caused by a shotgun. Patients with private insurance had lower mortality odds than those with Medicare/Medicaid (OR 2.4, 95% CI 1.7–3.4, p < 0.001) or government insurance (OR 3.6, 95% CI 2.2–5.8, p < 0.001). Management at level II centers, compared to level I, was associated with lower odds of returning home (OR 0.3, 95% CI 0.2–0.5, p < 0.001)

**Douglas & Rogers, 2024[14]**	To elevate the experiences of two healthcare providers	Autoethnography (Qualitative)	Staff, residents, and faculty in trauma centers bear the brunt of this trauma, second only to the families and communities that suffer the loss of loved ones. This burden is especially heavy for health care workers who share the same ethnic background of those who are disproportionately affected by interpersonal gun violence.
**Felix et al., 2018[15]**	To explore students' psychosocial adjustment following a mass murder tragedy, what changed or did not change from their pre-tragedy adjustment levels, and their view on what helped most in the immediate aftermath	Prospective Cohort Study (Quantitative)	Students with any resource loss had a steeper incline in symptoms than did students reporting no resource loss. From pre- to post-tragedy, there was an increase in psychological sense of school membership but no change in general self-efficacy and social support. Students with clinical levels of posttragedy distress reported more childhood trauma and depression symptoms at college entry. Student-initiated and -led memorial activities were rated as most helpful.
**Fergus et al., 2011[16]**	To explore subjective and objective reactions to writing and reading a narrative of their experience to explore PTS, depression, anxiety symptoms, and physical exposure after having been recently exposed to a campus shooting	Longitudinal Study (Quantitative)	Post-traumatic stress symptoms emerged as the most robust predictor of distress. Psychological symptoms, but not physical exposure, tended to correlate significantly with subjective distress at post-writing and post-reading.
**Greenspan & Kellermann, 2002[17]**	To determine the health status and psychological distress of gunshot injury victims 8 months after hospital discharge	Cohort Study (Quantitative)	Many hospitalized survivors of gunshot injury still experience considerable physical and psychological morbidity 8 months after discharge.
**Grills-Taquechel et al., 2011[18]**	To examine the influence of a mass trauma (the Virginia Tech campus shootings) on anxiety symptoms and quality of life, as well as the potential vulnerability/protective roles of world assumptions and social support	Cohort Study (Quantitative)	The experience of this mass trauma did not generally alter the anxiety levels or QoL of women enrolled at the university where it occurred. However, greater belief in a lack of control over outcomes was significantly associated with increased physiological/emotional arousal anxiety symptoms for those more severely exposed to the shooting. Our findings show that, even after a traumatic event, those with higher self-worth beliefs tended to report better psychosocial adjustment.
**Hawkins et al., 2004[19]**	To highlight similarities and variability in immediate emotional, cognitive, and social responses to mass violence	Qualitative	There exists important variation in responses during the earliest periods after a traumatic event. Negative emotions were not uniformly more prominent than positive ones; indeed the most intense emotion reported was affection. Regarding social support, respondents noted that talking and being with friends and family was most often helpful. However, being asked or forced to talk was perceived by several as harmful.
**Herrera-Escobar et al., 2021[20]**	To assess outcomes in survivors of firearm injuries after 6 to 12 months and compare them with a similarly injured trauma population	Prospective Cohort Study (Quantitative)	Among firearm injury survivors, 67.7% reported daily pain, 53.2% screened positive for PTSD, 38.7% reported a new functional limitation in an activity of daily living, and 59.1% have not returned to work. Compared with matched MVC survivors (n = 255), firearm injury survivors were significantly more likely to have daily pain [adjusted odds ratio (OR) 2.30, 95% confidence interval (CI) 1.08–4.87], to screen positive for PTSD (adjusted OR 3.06, 95% CI 1.42–6.58), and have significantly worse physical and mental health-related quality of life.
**Hink et al., 2022[21]**	To explore prior violent exposures, risks, recovery, supportive services, outcomes, and views of firearms and violence among survivors of firearm assaults and unintentional injuries	Qualitative	Given that most individuals who were assaulted did not identify a personal risk that directly contributed to them being victimized, they felt there were larger societal contributors to firearm violence that need to be addressed to reduce the "roots" of firearm violence such as education and social safety net programs that aim to support high-risk youth. They universally noted the importance of their family and social networks, and having financial safety-nets through health insurance, victims of crime assistance, and other financial assistance programs.
**Holloway et al., 2023[22]**	To examine the impacts of gun violence on early childhood development including early childhood mental health, cognitive development, and the assessment and treatment of survivors	Literature Review	Exposure to gun violence is associated with an increase in children's mental health distress, with young children experiencing increased post-traumatic stress symptoms and anxiety
**Hootman et al., 2000[23]**	To characterize non-fatal firearm related injuries other than gunshot wounds (non-GSWs) treated in hospital emergency departments that occur during routine gun handling and recreational use as well as violence-related use of a firearm	Cross Sectional Study (Quantitative)	Non-fatal, non-GSWs make a notable contribution to the public health burden of firearm related injuries. Firearm related injury prevention programs should focus on not only the reduction of gunshot wounds but also the reduction of unintentional and violence related non-GSWs.
**Hureau et al., 2022[24]**	To assess the extent to which violence intervention workers experience Secondary Traumatic Stress (STS)	Cross Sectional Study (Quantitative)	Across three work-related traumas, 105 workers reported having seen someone get shot at while on the job, 34 workers reported ever having gotten shot at while on the job, and 91 workers reported having experienced the death of a client due to violence. Experiencing addi-tional work-related traumas increases STS; and the death of a client due to violence increases STS regardless of whether an interventionist witnessed the shooting.
**Johnson et al., 2002[25]**	To examine the longitudinal course of psychiatric sequelae of a mass shooting incident at a courthouse	Cohort Study (Quantitative)	Only 5% of the study sample met criteria for PTSD after this incident. Universal distress, however, was evident as 96% of the respondents reported PTSD symptoms and 75% described the incident as "very upsetting." The need for intervention among symptomatic individuals not meeting diagnostic criteria should not be discounted as subdiagnostic distress may warrant specific intervention.
**Jones 2020[26]**	To share experiences as a pediatric trainee advocate for gun violence prevention while amplifying the voices of survivors	Qualitative	Each day, ~210 people go on to survive their gun injuries; that's 76,000 survivors every year. Attention to survivors is not only critical in terms of their own healing, but also in terms of their wealth of lived experience, which could play a pivotal role in bringing about solutions

**Joseph et al., 2019[27]**	To examine the effect of different types of firearms on readmission due to acute stress disorder (ASD) and/or post-traumatic stress disorder (PTSD) in firearm-injury victims	Retrospective Cohort Study (Quantitative)	Victims of semiautomatic rifles were more likely to be men, aged between 18 and 44 years, in the lowest median household income quartile; they are more likely to have been assaulted in comparison to shotgun injury patients. Victims of shotgun-related injuries were less likely to be male, but were more likely to abuse alcohol, and have preinjury psychiatric disorders. Victims of shotgun injuries were more likely to have self-inflicted injuries. On univariate analysis, patients with shotgun-related injuries had the highest rate of readmission with ASD/PTSD (16%) followed by semiautomatic rifle–injured patients (9%) and handgun injury patients (7%) (P < 0.001).
**King & Huang, 2024[28]**	To explore relationships between gun violence and the risk of housing instability, examining how this may be mediated by other neighborhood social and physical factors	Retrospective Cohort Study (Quantitative)	Women who witnessed violence are more likely to experience physical and mental health problems with direct associations being 0.06 and 0.042, and those in poor physical and mental health in turn are at higher risk of experiencing housing instability with direct associations being 0.065 and 0.155, respectively.Those who have deadly gun violence near their home are more likely to have lower household income (direct association = −0.311) and lower level of social support albeit only indirectly (indirect association = −0.061)
**Kwon et al., 2023[29]**	To describe interpersonal violence-related injury patterns in the pediatric trauma population and to identify predictors of recidivism	Retrospective Cohort Study (Quantitative)	Survivors of firearm injuries and assault comprise a vulnerable patient cohort at risk for repeat injury, and Black race is an independent predictor of repeat injury owning to IPV. Black race and firearm injury were associated with more than 3-fold higher likelihood of repeat injury compared to white race after adjusting for age, sex, insurance, and child opportunity index
**Lal et al., 2023[30]**	To describe the patterns of Firearm-Related Injuries (FRI) in the United States during the period of 2016–2019 and to evaluate the patient-centered outcomes in the survivor (non-fatal injuries) versus the non-survivor (fatal injuries) groups	Cohort Study (Quantitative)	The individuals from the lowest quartile of annual household income, males, young Americans, and racial minorities were disproportionally affected. The non-survivor (fatal injuries) group had a shorter length of stay in the hospital by 5.1 days (95% CI − 5.64 to − 4.58, p value= <0.01), the higher median cost of hospitalization by $8903 (95% confidence interval $311.9 to $17,494.2, p value=0.04), and a higher median cost of hospitalization per day by $41,576.74 (95% confidence interval $40,333.1 to $42,820.3, p value= <0.01).
**Leibbrand et al., 2021[31]**	To explore whether exposure to local gun violence is associated with higher risks of depression among mothers, whose symptoms of depression are likely to have spillover effects for kin	Cohort Study (Quantitative)	Mothers who witness at least one shooting in their neighborhoods or local communities exhibit more symptoms of depression and are 32–60% more likely to meet criteria for depression than mothers who do not witness a shooting. Witnessing a shooting is also associated with increases in parental aggravation, which is partially mediated by maternal depression.
**Lennon et al., 2024[32]**	To describe how often Chicago children are exposed to firearm violence, the types of exposure, and the parent-reported impact of these exposures on child mental health symptoms	Cross Sectional Study (Quantitative)	More than one third (37%) of children were exposed to firearm violence with an indirect exposure prevalence of 32% and a direct exposure prevalence of 10%. Mental health symptoms associated with firearm violence exposure were reported for 20% of children. Mental health symptoms were reported for 7% of children without firearm violence exposure compared to 31% with indirect exposure and 68% with direct exposure.
**Liu & Kia-Keating, 2019[33]**	To determine classes of exposure to an event of mass violence, and if posttrauamtic stress syndrome (PTSS) differed across classes	Retrospective Cohort Study (Quantitative)	4 categories of exposure were classified: minimal exposure (55.5% of the sample), auditory exposure (29.4%), visual exposure (10%), and interpersonal exposure (5%). More severe direct exposure (i.e., the visual exposure class) was associated with significantly higher levels of PTSS. There were no significant differences in PTSS between the auditory exposure and minimal exposure classes.
**Lumbard et al., 2023[34]**	To characterize those with and without readmissions, identify risk factors for 90-day unplanned readmission, and examine reasons for hospital readmission	Retrospective Cohort Study (Quantitative)	There were no significant differences in age or payor, but more women (14.7% vs. 23%) and older children (13–17 years [80.5%]) had readmissions. The mortality rate during primary hospitalization was 5.1%. Survivors of initial firearm injury were more frequently readmitted if they had a mental health diagnosis. Readmission diagnosis included complications (15%), mental health or drug/alcohol (9.7%), trauma (33.6%), a combination of the prior three (28.3%), and chronic disease (13.3%). Female children, those with longer lengths of stay, and those with more severe injuries were more likely to have unplanned 90-day readmissions.
**Magee et al., 2022[35]**	To understand the mental health outcomes and needs of nonfatal firearm violence survivors and their family members	Cohort Study (Quantitative)	Mental health prevalence rates increased by nearly 3% for family members of nonfatal shooting sur-vivors in the 12-months following a nonfatal shooting, compared to the preinjury period. Among youth with a new mental health diagnosis over half were family members and no differences were observed in mental health conditions between survivors and family members
**Magee et al., 2023[36]**	To determine facilitators and barriers to mental health care engagement among firearm injury survivors	Qualitative	Survivors described family members, friends, and informal networks as their main source of emotional support. Barriers to mental health care utilization were perceived lack of benefit, distrust in practitioners, and fear of stigma. Credible messengers served as facilitators to mental health care. Survivors also described the emotional impact their shooting had on their families, particularly mothers, partners, and children.
**Mancini et al., 2016[37]**	To examine the possibility that mass trauma can promote psychological improvement for some survivors	Prospective Cohort Study (Quantitative)	Although most individuals showed a resilient response (56% to 59%), displaying low levels of anxiety and depression both before and after the shooting, some exhibited chronic distress and others a pattern of continuous high distress. Some participants showed substantial improvements in depression and anxiety; these individuals had elevated levels of depression and anxiety before the shooting and experienced a marked reduction soon after. The improvement pattern was associated with substantial increases in perceived social support and gains in social resources, as predicted by the idea that acute stress promotes stronger social relationship.
**McCall, 2020[38]**	To learn of emergency nurses' experiences of caring for patients from a school shooting event in an effort to benefit future preparedness, response, and recovery	Case Series (Qualitative)	The presence of vivid recollections 18 months after providing care to the patients from the multiple-victim, school-associated shooting are reported. Some participating emergency nurses also discussed thought intrusions when not in the clinical or work setting. Results suggest the importance of having protective mechanisms for coping while working with trauma patients.

**McGee et al., 2017**[39]	To explain youth delinquency, namely committing a crime with a gun, as an effect of exposure to violence and peer victimization	Cross Sectional Study (Quantitative)	Direct victimization, as a measure of exposure to violence, was the best predictor of problem behavior as measured by gun related delinquency, particularly for males. Results indicate the importance of continued examination of community and school based preventions focusing on the specific needs of students exposed to danger.
**McLaughlin & Kar, 2019**[40]	To evaluate the clinical presentation of post-traumatic stress disorder (PTSD) in an adolescent by highlighting the clinical course of a school shooting survivor	Case Report (Qualitative)	Clinicians need to be able to recognize and treat symptoms of PTSD in adolescent patients. In addition to appropriate medications and psychotherapy, the clinician needs to be prepared to face barriers to remission such as re-exposure through media content. This case study also prompts physicians to recognize avoidance behaviors of such social media and news content as a manifestation of PTSD in adolescents.
**Mitchell et al.,2021**[41]	To examine the impact of seeing and hearing gun violence on youth of different ages, living in urban and non-urban areas	Cross Sectional Study (Quantitative)	41% of youth in this study reported ever seeing or hearing gun violence; 32% had such an experience in the past year. Among exposed youth, 50% took protective action to keep themselves safe, and 58% reported being very or extremely afraid, sad, or upset as a result of the indirect gun violence. More youth living in urban compared with non-urban areas took some protective action. Females and younger children had increased odds of experiencing high fear as a result of the violence.
**Molina et al., 2019**[42]	To explore factors affecting community resilience from the perspective of social service providers, mental health practitioners, and community leaders who were first responders during the Pulse Nightclub attack	Case Series (Qualitative)	Six themes were eluciated: (1) the attack was a hate crime and an act of terrorism targeted at LGBTQ Latinx, (2) the community grew stronger and closer and became resilient subsequent to the attack, (3) linguistic and cultural competence were critical to responding to this act of terrorism, (4) survivors are still hurting and traumatized one year later, (5) there is a need for long-term mental health services and a plan for dealing with mass shootings and terrorist attacks, (6) and important lessons learned from this event may be helpful in developing community resilience
**Molnar et al., 2004**[43]	To identify features of neighborhoods associated with concealed firearm carrying among a representative sample of youth from Chicago, Illinois	Cross-Sectional Study	One third (32%) of respondents reported that they had a family member who had been shot with a gun, and these accounted for 76% of participants who carried concealed firearms. Of the 56 youth who reported having carried a concealed gun, most had been exposed to high levels of violence: 93% were in the highest quartile on the scale of witnessing violence in the past year, 63% were in the highest quartile of having heard about violent victimization of acquaintances during the past year, and 91% were in the highest quartile reporting victimization by violence in the previous year.
**Mooney, 2013**[44]	To explore the psychological and professional effects on teachers witnessing an episode of severe school violence	Qualitative (Dissertation)	In the face of school shootings, sense of security at school is lost; teacher turnover in many states is high; these shootings affect people psychologically, and often permanently; impact communities beyond the school; the impacts of school shootings vary greatly, and often determined by a number of variables, such as the intensity of the incident, the background of the educator involved, and the handling of the situation by authorities
**Mueller et al., 2023**[45]	To examine the incidence and factors associated with recurrent firearm injuries and death among patients presenting with an acute (index), nonfatal firearm injury	Retrospective Cohort Study (Quantitative)	Recurrent injury and death are frequent among survivors of firearm injury, particularly among patients from socially vulnerable areas. Over a median follow-up of 3.5 years (IQR, 1.5 to 6.4 years), 1155 patients experienced a recurrent firearm injury including 5 firearm suicides and 149 fatal firearm injuries. Persons experiencing recurrent firearm injury were young (25.3 ± 9.5 years), predominantly male (93%), Black (96%), and uninsured (50%), and resided in high social vulnerability regions (65%). The estimated risk for firearm re-injury was 7% at 1 year and 17% at 8 years.
**Nehra et al., 2021**[46]	To describe the demographic, injury-related, and mental health characteristics of firearm injury patients and trace firearm weapon carriage and PTSD symptoms over the year after injury	Prospective Cohort Study (Quantitative)	Firearm injury survivors are at risk for firearm carriage and high PTSD symptom levels post-injury. The significant decrease in the high-risk behavior of firearm weapon carriage at 3–6 months post-injury suggests that there is an important post-injury ''teachable moment'' that should be targeted with preventive interventions.
**North et al., 2002**[47]	To examine the course of post-disaster posttraumatic stress disorder (PTSD) and major depression in individuals 3 years after a mass shooting episode	Retrospective Cohort Study (Quantitative)	Female gender was found to be associated with recovery. Index symptoms of any symptom group not found to predict outcome, even though other studies have observed early PTSD symptoms to be predictive of the course of PTSD. Those who recovered from PTSD reported fewer symptoms over time in all three symptom groups, but those who did not demonstrated increasing numbers of avoidance and numbing symptoms over time.
**O'Neill et al., 2020**[48]	To characterize the post-hospitalization recovery experience of survivors of gun violence	Community-based Participatory Research (Qualitative)	Five recurring themes emerged: (1) Isolation, physical and social restriction due to fear of surroundings; (2) Protection, feeling unsafe leading to the desire to carry a gun; (3) Aggression, willingness to use a firearm in an altercation; (4) Normalization, lack of reaction driven by the ubiquity of gun violence in the community; and (5) Distrust of health care providers, a barrier to mental health treatment. Survivors of gun violence describe a disrupted sense of safety following their injury. As a result, they experience isolation, an increased need to carry a firearm, a normalization of gun violence, and barriers to mental health treatment. These maladaptive reactions suggest a mechanism for the violent recidivism seen among survivors of gun violence and offer potential targets to help this undertreated, high-risk population.
**O'Neill et al., 2022**[49]	To evaluate the risk of repeat firearm injury in survivors of firearm violence in Connecticut	Retrospective Cohort Study (Quantitative)	Of the patients presenting with an initial FRI in the YNHH system, one in twelve will experience another firearm injury within the next 5 years. Of the 94 patients, 8.5% (8) had a repeat firearm related injuries (FRI) and 2% (2) died from homicide within the next 5 years. Compared with nonviolence-related trauma patients from 2014 (n =2001), those with an FRI had 12 times the odds of a repeat firearm injury (odds ratio: 12.0, P = 0.047) in the next 5 years after adjustment for relevant covariates.

**Oddo et al., 2021[50]**	To quantify new mental health diagnoses after non-fatal firearm injury	Retrospective Cohort Study (Quantitative)	Over a quarter of youth with nonfatal firearm injury were diagnosed with a new mental health condition in the 12 months after their injury. Medicaid insurance and a prior complex chronic condition were predictors of new mental health diagnosis.
**Orlas et al., 2021[51]**	To describe the current literature regarding long-term physical, mental, and social outcomes of firearm injury survivors in the United States	Scoping Review (Mixed Methods)	Research on the long-term health impact of firearm injury is scant, and heterogeneity in available studies limits the ability to fully characterize the outcomes among these patients. A better understanding of the long-term health impact of firearm injury would support systematic change in policy and patient care to improve outcomes.
**Phillips et al., 2020[52]**	To describe the incidence, injury severity, and institutional costs of pediatric gun-related injuries in Colorado	Retrospective Cohort Study (Quantitative)	The overall mortality rate was 11% (34/308), with significantly fewer children (5%) dying from their injuries when compared with adolescents (14%; p = 0.04). Sixty-five (21%) patients required blood product transfusions, with 23 (7.4%) patients receiving a massive transfusion. Overall, 52% (161/308) required a major operation, with 15% undergoing an exploratory laparotomy. One third (4/13) of the patients who had a thoracotomy in the emergency department survived to hospital discharge. Overall, 14.0% of patients had psychiatric follow-up at both 30 days and 1 year. The readmission rate for complications was 11.6% at 30 days and 14% at 1 year. The total cost of care for all pediatric firearm-related injuries was approximately US $26 million.
**Piskorik 2022[53]**	To explore how students have been influenced by their experience surviving the 2018 Marjory Stoneman Douglas High School shooting during their transition to and performance in higher education	Qualitative (Dissertation)	Trauma, the role of advocate, and identity are uniquely connected for these survivors of gun violence. Situation, sense of self, support, and strategies for coping were crucial elements for each students' transition into college - academically, socially, physically, and geographically.
**Pulcini et al., 2021[54]**	To compare mental healthcare encounters and expenditures of youth preceding and folllowing a nonfatal firearm injury	Retrospective Cohort Study	For the high prior mental healthcare expenditures, there was a 44% decrease in mental healthcare encounters (pre=13,394, post=7,527, p<0.001), which accounted for most of the overall decrease in mental healthcare encounters. Those with high non-mental health expenditures had a 18% decrease in mental healthcare encounters (pre=2,914, post=2,389, p=0.031). There was a decrease of $346,029 in mental health expenditures overall, an increase of $804,024 in the healthy subgroup, a >$1 million decrease among the high prior mental health expenditure subgroup, and a $75,000 decrease in the high non-mental health expenditure group. All of the expenditure changes were statistically significant (p<0.001).
**Richardson et al., 2020[55]**	To explore how survivors of gun violence express symptoms of traumatic stress and the ways in which their narratives can inform the implementation of the biopsychosocial model in HVIPs	Phenomenological (Qualitative)	Symptoms of traumatic stress, altered relationships to others, and new forms of meaning making (e.g., spirituality) demonstrate the relationships between psychological and social impacts of violence. The young men in the study illuminated nuanced narratives of their coping strategies, meaning-making processes, and personal health-related goals.
**Richmond & Lemaire, 2008[56]**	To estimate the life expectancy loss attributable to the premature death of individuals who initially survived gun-related traumatic brain injury (TBI) and spinal cord injury (SCI) in the United States	Retrospective Cohort Study (Quantitative)	On average, across age, gender, and race, life expectancy in the United States is reduced by 3.1 days because of the shorter lifespan for individuals who survive an initial gunshot wound to the brain or spinal cord. Black males bear a disproportionate burden, losing 9.5 days, whereas white males lose 4.6 days. Black and white females lose 1.5 and 1.0 days, respectively. This results in a total of 106.7 days of life lost from gunshot wounds for the general U.S. population, with 371.0 days of life lost for black males.
**Rivara et al., 2023[57]**	To quantify non-fatal firearm injuries and the characteristics of patients and injuries	Cross-Sectional Study (Quantitative)	Firearm injured patients living in rural areas were somewhat older than those in urban areas. They were much less likely to be Black compared to patients in urban areas (18.4% vs 83.9%) Patients living in urban areas were more likely to be uninsured. Three-quarters of patients in urban areas were injured in assaults compared with less than half in rural areas. Fewer patients in urban areas received any post-discharge services. Only 12.3% of patients received services on discharge from the hospital; only 4.3% received social work or mental health services and only 5.4% received any services related to violence.
**Rosas et al., 2021[58]**	To perform an epidemiological evaluation and an economic analysis of 90-day costs associated with non-fatal gunshot wounds (GSWs) to the extremities, spine and pelvis requiring orthopaedic care in the United States	Retrospective Epidemiological Review (Quantitative)	Of 2183 fractures due to GSW, 22% were femur fractures, 18.3% were hand/wrist fractures and 16.7% were ankle/foot fractures. A majority of patients were male (83.3%) and under 65 years of age (56.3%). Total charges for GSW requiring orthopedic care were $513,334,743 during the 10-year study period. Total reimbursement for these patients were $124,723,068. Average charges per patient were highest for fracture management of the spine $431,021.33, followed by the pelvis $392,658.45 and later by tibia/fibula fractures $342,316.92. Nonfatal gunshot wounds have large economic and societal impacts - 90 day direct charges and reimbursement of orthopedic care are significant per patient.
**Rowhani-Rahbar et al., 2015[59]**	To compare the risk for subsequent violent injury, death, or crime perpetration among patients with an firearm-related hospitalization (FRH), those hospitalized for non-injury reasons, and the general population	Retrospective Cohort Study (Quantitative)	Among patients with an index FRH (n = 613), rates of subsequent FRH, firearm-related death, and firearm- or violence-related arrest were 329 (95% CI, 142 to 649), 100 (CI, 21 to 293), and 4221 (CI, 3352 to 5246) per 100,000 person-years, respectively. Compared with the general population, standardized incidence ratios among patients with an index FRH were 30.1 (CI, 14.9 to 61.0) for a subsequent FRH and 7.3 (CI, 2.4 to 22.9) for firearm-related death. Patients with an index FRH were at greater risk for subsequent FRH (subhazard ratio [sHR], 21.2 [CI, 7.0 to 64.0]), firearm-related death (sHR, 4.3 [CI, 1.3 to 14.1]), and firearm- or violence-related arrest (sHR, 2.7 [CI, 2.0 to 3.5]) than those with a non–injury-related index hospitalization.
**Ryan & Hawdon, 2008[60]**	To discuss what individuals personally experienced on 4-16 and what was done as faculty members of the sociology department in the first few weeks after 4-16	Case Study (Qualitative)	Four main takeaways: (a) The tragedy is defined by members as affecting the collective, (b) the tragedy is a "fateful event" that disrupts the routine of everyday life, (c) the collective is seen as an unwilling participant in the tragedy, and (d) the collective is seen as a "moral collective."

**Sabri & Campbell, 2023[61]**	To highlight the consequences and barriers commonly faced by immigrant women in abusive relationships with presence of a firearm as identified by survivors and service providers	Qualitative	Participants highlighted impacts of exposure in terms of living in constant fear or threat of being hurt, chronic stress and various physical and mental health challenges related to repeated firearm presence due to prior exposures to firearms and legal, financial, and immigration-related consequences.
**Sarani et al., 2021[62]**	To describe causes of death in victims of civilian public mass shootings (CPMSs) as well as the injuries sustained and treatments rendered in survivors to inform ideal preparation for these events	Retrospective Cohort Study (Quantitative)	Median number of patients seen per event was 20, distance to each hospital was 6 miles, time to arrival was 56 minutes, number of wounds per patient was 1, and Injury Severity Score was 5. Most common injuries were extremity fracture (37%) and lung parenchyma (14%). Following arrival to the hospital, 27% were discharged from the emergency department, 32% were taken directly to the operating room/interventional radiology, 16% were admitted to the intensive care unit, and 25% were admitted to the ward. Forty percent did not require advanced treatment within 12 hours. Most common operations performed within 12 hours of arrival were orthopedic (15%) and laparotomy (15%). The most common specialties consulted were orthopedics (38%) and mental health (17%).
**Semenza & Stansfield, 2021[63]**	To examine the relationship between fatal and non-fatal gun violence and rates of functional disability among men	Cross Sectional Study (Quantitative)	High rate shooting neighborhoods have substantially higher than average rates of all functional disabilities; males ages 18 to 64 have more than double the percentage of all disability types in high shooting rate neighborhoods compared to low rate neighborhoods; social isolation, chronic illness, unhealthy behavior, and poverty were consistently associated with higher prevalence of community disability irrespective of age.
**Semenza & Stansfield, 2021[64]**	To examine the association between total, fatal, and non-fatal gun violence and measures of physical inactivity, obesity, sleep, and smoking	Cross Sectional Study (Quantitative)	A higher total rate of shootings is significantly associated with the composite measure of unhealthy behaviors in neighborhoods (B = 0.081, SE = 0.021, p<0.001). The rate of fatal shootings is not statistically associated with poor health behaviors whether entered into the model individually or simultaneously with the non-fatal shooting rate.
**Semenza et al., 2024[65]**	To evaluate whether gun violence exposures (GVEs) are associated with suicidal ideation and behaviors among Black adults	Cross Sectional Study (Quantitative)	Being threatened with a gun (odds ratio [OR], 1.44; 95% CI, 1.01-2.05) or knowing someone who has been shot (OR, 1.44; 95% CI, 1.05-1.97) was associated with reporting lifetime suicidal ideation. Being shot was associated with reporting ever planning a suicide (OR, 3.73; 95% CI, 1.10-12.64). Being threatened (OR, 2.41; 95% CI, 2.41-5.09) or knowing someone who has been shot (OR, 2.86; 95% CI, 1.42-5.74) was associated with reporting lifetime suicide attempts. Cumulative GVE was associated with reporting lifetime suicidal ideation (1 type: OR, 1.69 [95% CI, 1.19-2.39]; 2 types: OR, 1.69 [95% CI, 1.17-2.44]; 3 types: OR, 2.27 [95% CI, 1.48-3.48]), suicide attempt preparation (3 types; OR, 2.37; 95% CI, 2.37-5.63), and attempting suicide (2 types: OR, 4.78 [95% CI, 1.80-12.71]; 3 types: OR, 4.01 [95% CI, 1.41-11.44]).
**Shockley, 2023[66]**	To examine how relatives of gun-violence victims, specifically Black women, move about their environments in the aftermath of sudden and tragic loss	Black Feminist Auto-Ethnography (Qualitative)	The disconnect between law enforcement and our co-victims makes the grieving process more difficult because survivors are left without information, support, and closure with only word of mouth confirmation from the streets about the perpetrator. Social networks were vital in our grieving and healing process. Family, friends, neighbors, and the church offered places of solace.
**Sillito & Salari, 2011[67]**	To examine outcomes of children in households that experience intimate partner homicide-suicide	Retrospective Cohort Study (Quantitative)	The majority of children witnessed the IPHS (52.8%), and there were about equal proportions of children killed (23.2%), and not present (23.9%). ~14% of children in the sample called emergency services for their parents and/or discovered the bodies. In 76.64% of cases, the children were biological children of both the perpetrator and the victim. Children in cases with homicidal-intent perpetrators were most likely to witness an IPHS (63.7%); children with suicidal-intent perpetrators were most likely to be killed.
**Smith et al., 2018[68]**	To study the psychological risks associated with retained bullets	Prospective Cohort Study (Quantitative)	Of those working prior to injury (n=47), 61% with retained bullets had not returned to work compared to 33% without retained bullets (p=0.027). No difference in PCL-5 scores [30.9 (SD 18.9) vs 27.9 (SD 18.6), p=0.470] was observed, but patients with retained bullets had greater mean QIDS-SR scores [10.7 (SD 6.2) vs 7.8 (SD 6.1), p=0.038] than those without. After controlling for injury severity, number of wounds, marital status and education level, multiple linear regression analysis determined that retained bullets (β=3.52; p=0.017) were associated with more severe depressive symptoms.
**Smith et al., 2020[69]**	To provide a detailed analysis of injuries, responses, and need to guide hospitals who strive to prepare for future similar events	Retrospective Cohort Study (Quantitative)	Of the 102 victims, 40 died at the scene and 9 died upon arrival to the Level I trauma center. The remaining 53 victims received definitive medical care and survived. Twenty-nine victims were admitted to the trauma center and five victims to a community hospital. The remaining 19 victims were treated and discharged that day. Decedents sustained significantly more bullet impacts than survivors (4 ± 3 vs. 2 ± 1; p = 0.008) and body regions injured (3 ± 1 vs. 2 ± 1; p = 0.0002). Gunshots to the head, chest, and abdominal body regions were significantly more common among decedents than survivors (p < 0.0001). Eighty-two percent of admitted patients required surgery in the first 24 hours. Essential resources in the first 24 hours included trauma surgeons, emergency room physicians, orthopedic/hand surgeons, anesthesiologists, vascular surgeons, interventional radiologists, intensivists, and hospitalists.
**Smith et al., 2023[70]**	To explore the psychological impacts of retained bullet fragments (RBFs) on individuals who have experienced recent firearm-related injuries (FRI)	Ethnography (Qualitative)	Retained bullet fragment-associated pain caused sleep disturbances, limited mobility, and inability to exercise or complete activities of daily living. Participants experienced anger, fear, shame, regret, pride, and vulnerability, unpredictable mood changes, flashbacks, nightmares, paranoia, hypervigilance, and clinical diagnoses of post-traumatic stress disorder (PTSD). They also described relying more on family and friends after their FRI, which was especially true for those with limited mobility due to their injury. Many were unable to work for a period of time following their injury - not only a financial stressor but also lack of bringing meaning and purpose to their lives; some described RBF as an inspiration to work harder, go back to school, or set higher goals for themselves.

**Smith, 2020**[71]	To explore mass school shootings and how they affect secondary public-school youth who are not within the immediate geographic area of the shootings	Qualitative (Dissertation)	Seven themes emerged: early interventions to address increased stress on students are crucial; there is an increased awareness of firearm violence resulting from media accessibility; there's an increased sense of safety when practicing active shooter drills; one-to-one sessions vs group sessions aid in combatting student stress; rapport building is critical in trauma-informed counselling; mixed feelings of a need for increased security and a lack of awareness about security concerns emerge among students; school shootings and increased awareness of them can correspond to feelings of uncertainty, fear, irritation, and agitation, prompting acting-out behaviors.
**Song et al., 2022**[72]	To measure changes in clinical and economic outcomes after nonfatal firearm injuries among survivors and their family members	Cohort Study (Quantitative)	After nonfatal firearm injury, medical spending increased $2495 per person per month (402%) and cost sharing increased $102 per person per month (176%) among survivors relative to control participants (P < 0.001) in the first year after injury, driven by an increase in the first month of $25 554 (4122%) in spending and $1112 (1917%) in cost sharing per survivor (P < 0.001). Survivors had a 40% increase in pain diagnoses, a 51% increase in psychiatric disorders, and an 85% increase in substance use disorders after firearm injury relative to control participants (P < 0.001), accompanied by increased pain and psychiatric medications. Family members had a 12% increase in psychiatric disorders relative to their control participants (P = 0.003). These overall clinical and economic changes were driven by intentional firearm injuries and more severe firearm injuries
**Song et al., 2023**[73]	To understand the clinical and economic impact of firearm injuries and family members	Cross Sectional Study (Quantitative)	Through one year after firearm injury, child and adolescent survivors experienced a 117 percent increase in pain disorders, a 68 percent increase in psychiatric disorders, and a 144 percent increase in substance use disorders relative to the controls. Survivors' health care spending increased by an average of $34,884—a 17.1-fold increase—with 95 percent paid by insurers or employers. Parents of survivors experienced a 30–31 percent increase in psychiatric disorders, with 75 percent more mental health visits by mothers, and 5–14 percent reductions in mothers' and siblings' routine medical care. Family members of decedents experienced substantially larger 2.3- to 5.3-fold increases in psychiatric disorders, with at least 15.3-fold more mental health visits among parents.
**Spearman et al., 2023**[74]	To provide insight from maternal survivors of intimate partner violence (IPV) describing their experiences with their ex-partners' firearm ownership, access, storage and behaviors in the context of co-parenting and separation	Qualitative	Six themes emerged related to firearm experiences and post-separation abuse: (1) gun ownership (2) gun access; (3) unsafe storage; (4) direct and symbolic threats; (5) involving the children; (6) survivors' protective actions. This provides context on how abusive ex-partners' firearm ownership, access and threats cause terror and pervasive fear for mothers and children following separation.
**Spinuzzi, 2023**[75]	To explore how survivor perception affects trauma response after exposure to a mass shooting	Qualitative (Dissertation)	Many themes emerge: disorientation during the event; trauma response and PTSD symptoms; negative long-term adjustments; positive changes in lifestyle and worldview; isolation; individuals identification of the importance of having a support system that consisted of fellow mass shooting survivors
**Spitzer et al., 2022**[76]	To estimate the volume of, and hospital costs for, fatal and non-fatal firearm injuries from 2005 to 2015 for each region of the United States and analyze the proportionate cost by payer status	Cohort Study (Quantitative)	There were a total of 317,479 firearm related admissions over the study period: 52,829 (16.66%), 66,671 (21.0%), 134,008 (42.2%), and 63,972 (20.2%) for the Northeast, Midwest, South, and West respectively, demonstrating high regional variability. In the Northeast, hospital costs were $1.98 billion (13.9% of total), of which 56.0% was covered by government payers; for the Midwest, costs were $1.53 billion (19.7% of total), 40.4% of which was covered by government payers; in the South costs were highest at $3.2 billion (41.4% of total), but government payers only covered 34.3%; and costs for the West were $1.94 billion (25.0% of total), with government programs covering 41.6% of the cost burden.
**Turner et al., 2019**[77]	Across three work-related traumas, 105 workers reported having seen someone get shot at while on the job, 34 workers reported ever having gotten shot at while on the job, and 91 workers reported having experienced the death of a client due to violence. Experiencing addi-tional work-related traumas increases STS; and the death of a client due to violence increases STS regardless of whether an interventionist witnessed the shooting.	Cross Sectional Study (Quantitative)	Direct gun violence exposure, witnessing gun violence, and hearing gunshots were all significantly associated with other forms of victimization, rs = .10–.38, p < .001. For older youth (ages 10–17 years) polyvictimization was most strongly associated with posttraumatic symptoms, β = .35, p < .001, although witnessing gun violence still uniquely predicted a higher level of symptoms, β = .18, p < .01. For younger children (ages 2–9 years), hearing and witnessing gun violence were both related to posttraumatic symptoms, β = .15, p < .01 for both, even after controlling for polyvictimization.
**Vella et al., 2020**[78]	To determine the long-term functional, psychological, emotional, and social outcomes among survivors of firearm injuries	Prospective Cohort Study (Quantitative)	Combined alcohol and substance use increased by 13.2% (pre-GSW use, 56 [30.8%]; post-GSW use, 80 [44.0%]). Participants had mean (SD) scores below population norms (50 [10]) for Global Physical Health (45 [11]; P < .001), Global Mental Health (48 [11]; P = .03), and Physical Function (45 [12]; P < .001) PROMIS metrics. Eighty-nine participants (48.6%) had a positive screen for probable PTSD. Patients who required intensive care unit admission (n = 64) had worse mean (SD) Physical Function scores (42 [13] vs 46 [11]; P = .045) than those not requiring the intensive care unit. Survivors no more than 5 years after injury had greater PTSD risk (38 of 63 [60.3%] vs 51 of 119 [42.9%]; P = .03) but better mean (SD) Global Physical Health scores (47 [11] vs 43 [11]; P = .04) than those more than 5 years after injury

**Vicary & Fraley, 2010**[79]	To explore the impact of using the Internet for social-emotional purposes after school shootings	Retrospective Cohort Study (Quantitative)	Two weeks after the shooting, 71% of the respondents had significant depressive symptoms. Women reported more depressive symptoms than did men (M = 10.60, SD = 5.92), t(282) = 6.46, p < .001, d = .80. Participants who knew a victim reported more depressive symptoms than did those who did not know a victim (M =12.61, SD = 6.33), t(259) = 1.77, p = .006, d = .37. Women reported more PTSD symptoms than did men (M = 13.93, SD = 8.12), t(281) = 5.81, p < .001, d = .74. Participants who knew one of the victims (M = 19.78, SD =8.76) reported more PTSD symptoms than did those who did not know a victim (M = 16.48, SD = 9.04), t(258) = 2.82, p =.005, d = .37. 89% percent of participants had joined at least one Facebook group concerning the shooting. 28% percent left a message on the page of at least one of these groups. Data suggest that the use of the Internet had no beneficial or detrimental relationship to recovery—at least as far as depression and PTSD symptoms were concerned.
**Vinall, 2020**[80]	To explore the multiple lived experiences of mass shooting survivors through the lens of psychotherapists who have provided treatment subsequent to the trauma	Qualitative (Dissertation)	4 of 16 participants identified comorbid diagnoses present in some of their patients of pre-existing GAD, MDD, and personality pathologies. 8 of 16 participants specifically noted survivor guilt as a theme with which their clients struggled. Of the 13 participants who served as outpatient, non-crisis therapists, 7 identified vicarious trauma to family members as an outcome. Of the 13 participants who provided input to this question, 6 noted strengthening of clients' faith or spirituality, while 5 discussed clients questioning or wrestling with their faith. Major themes emerged in changes to primary relationships, vicarious trauma, and community fracture. 5 of 13 outpatient clinicians discussed the impact of agitated, jumpy, angry teachers in changing the "vibe" of the community.
**Wallace et al., 2023**[81]	To investigate the relationships between GV exposure, self-identified gender and perceptions of children's rights and safety	Cross Sectional Study (Quantitative)	Thirty-five percent reported witnessing GV. Fifteen percent of females reported never feeling safe at school compared to 3% of males (p=0.01). In multivariable logistic regression adjusted for gender, race/ethnicity and grade level, students who witnessed GV were 4.6 times more likely to report never feeling safe from violence (95% CI 1.7–12.4). Thirty percent of students who witnessed GV reported not attending school because of safety concerns. Students who witnessed GV had 2.2 times the odds of carrying a weapon to school (95% CI 1.1–4.5).
**Webb et al., 2024**[82]	To examine lived experiences of gunshot wound survivors while comparing different census categories and associated types of gunshot wound injuries	Mixed Methods (Interviews & Retrospective Review of EMR)	Those who self-identified as white had more self-inflicted GSWs than other groups. Those who self-identified in the minority population had more GSWs as a result of an altercation or as an innocent bystander. Of the 96 participants interviewed, 21 participants owned a gun at the time the GSW occurred, 47% and 15% of the White and Minority populations, respectively, p=.002. 49% of all participants, faced other struggles since the time of their GSW including: Trauma/PTSD/fear, Lost self/Depression, Functional Impairment/Pain, and Financial Struggles. When compared to the acute gunshot wound group (aGSW) the previous gunshot wound group (pGSW) responses showed support for increased gun safety and policies that limit firearm access.
**Weigend Vargas & Hemenway, 2021**[83]	To determine the effects of gun victimization on the likelihood of post emotional and physical symptoms as reported by victims	Cohort Study (Quantitative)	Roughly two-thirds (67%) of victims where the perpetrator used a gun reported suffering emotional symptoms compared to 59% of victims where the perpetrator used other weapons, and 52% were the perpetrator was unarmed. Victims were significantly more likely to report emotional and physical symptoms if injured, near the home, the crime involved multiple offenders, the victim was female, and the victim reported low household income.
**Wintemute et al., 2022**[84]	To assesses the prevalence and patterns of distribution of 6 experiences of violence (EVs) in an adult general population	Cross Sectional Study (Quantitative)	Gender was not associated with the prevalence of any experience. Non-owners of firearms who lived with owners reported more extensive EVs through social networks than did firearm owners or non-owners in households without firearms. Knowledge of people who had been shot by others was most common among Black respondents, 31.0% (95% CI 20.9–43.3%) of whom knew 2 or more such persons. More than 40% (42.8%, 95% CI 40.1–45.6%) of respondents encountered 1 or more sidewalk memorials in an average week; 1 in 5 (19.7%, 95% CI 17.5–21.9%) personally knew at least 1 person who had been "shot by someone else on purpose"; 1 in 7 (14.6%, 95% CI 13.0–16.5%) knew at least 1 person who had "shot themselves on purpose"; and approximately 1 in 8 knew at least 1 person they perceived to be at risk of violence to others (11.8%, 95% CI 10.1–13.7%) or to themselves (13.0%, 95% CI 11.2–15.0%).
**Zeineddin et al., 2020**[85]	To highlight the large toll firearm injuries take on American children, specifically in non-concealable disfigurements	Cross Sectional Study (Quantitative)	There were 8461 (29.6%) patients who underwent one of the major operations examined in this cohort. Open abdominal surgery was performed in 4565 (16.1%) and open reduction of long bones in 2866 (10%). Total mortality was 13% and 1500 (5.4%) were identified with one or more disfigurements: 220 amputations, 191 craniectomy, 100 enucleation, 533 ileostomy/colostomy, and 557 tracheostomies. Report highlights large toll that injuries take on American children, specifically in nonconcealable disfurements
**Zhao, 2021**[86]	To document how a family of Chinese descent spent their first 100 hours after the Atlanta Shooting on March 16, 2021 and how emotions, rooted in racialized lived experience and triggered by the mass shooting, evolved, shifted, and fueled the sentiments that gave rise to the Stop Asian Hate Movement	Autoethnography (Qualitative)	Mass shooting events impact communities with shared identities. "When do we turn our minor feelings into "major feelings"? At which point do we stop feeling stuck and denied? When do we feel empowered and hopeful again?" This personal, psychological impact sometimes turns into something collective and social - in this case, the Stop Asian Hate Movement.
**Zuo et al., 2018**[87]	To explore sex differences in short-term hospitalization outcomes after surviving firearm injury	Retrospective Cohort Study (Quantitative)	Among 17,594 males and 2,289 females discharged alive after index firearm injury hospitalization, 14.4% and 13.2% were readmitted within 180 days. Within 180 days, the risk of cardiovascular readmission was 3.3 times greater among males versus females (HR = 3.34, 95% CI [1.18, 9.44]. Risk of all-cause readmission among males was greater at 90 days (HR = 1.40, 95% CI [1.04, 1.87]. Firearm hospitalizations were mainly men compared to women (88.5% vs. 11.5%). Males were younger, more likely to be of assaultive intent, less likely to be suicidal, live in central and fringe metropolitan areas, have Medicaid (or self-pay/no charge), live in low-income neighborhoods and more likely to have fewer comorbidities than women. There was no difference in injury severity, cardiovascular comorbidity, or location by sex.
